# Supplementary material for: Solving the where problem and quantifying geometric variation in neuroanatomy using generative diffeomorphic mapping
Source: Nat Commun. 2025 Nov 24;16:10398. doi: 10.1038/s41467-025-65317-7 (PMC12645051; doi:10.1038/s41467-025-65317-7)
Supplement: Supplementary file 1 — Supplementary Information [file 41467_2025_65317_MOESM1_ESM.pdf]

Supplementary Note 1: Registration workflow

Registration workflow supplement

Supplementary note for Tward, et al. 2025

Introduction

In this supplement we summarize the steps that a user undertakes to register serial sections of a mouse brain to an annotated reference atlas.

Procedure

Data preparation: A brain id is provided by users and the location of the corresponding images is determined. A basic quality check is first performed automatically, including checking section number, image size, data structure, etc. If quality is as expected, and data ready to be registered, the high resolution images will be downsampled by a factor of 32 by 32, saved in TIF format, and sent to the registration pipeline.

Registration: The core of our registration code is written in python. It will first determine the modality of the brain images. Our pipeline is currently being applied to sections alternately imaged with Nissl and fluorescence signals, or Nissl and immunohistochemistry (IHC) staining. A simple initial slice alignment of Nissl images is applied by translating the center of mass of each section to the center of the image, followed by a rigid alignment of slices to a weighted combination of their nearest neighbors. An initial affine transformation is computed between the reference atlas and this initial stack, followed by deformable registration using the procedure outlined in the manuscript. Nissl images are rigidly registered to their adjacent images of the alternate modality. Transformation parameters (deformation fields, linear transformation matrices, scale change information) are output in VTK format (see the data format supplementary note), and several images are produced for quality control.

Registration quality control and correction: An online viewer was built for registration quality control (QC) purposes. During the registration step, a low resolution image with atlas overlay is generated for each section and these images are used for QC. An example of our QC interface is shown in Figure S1.

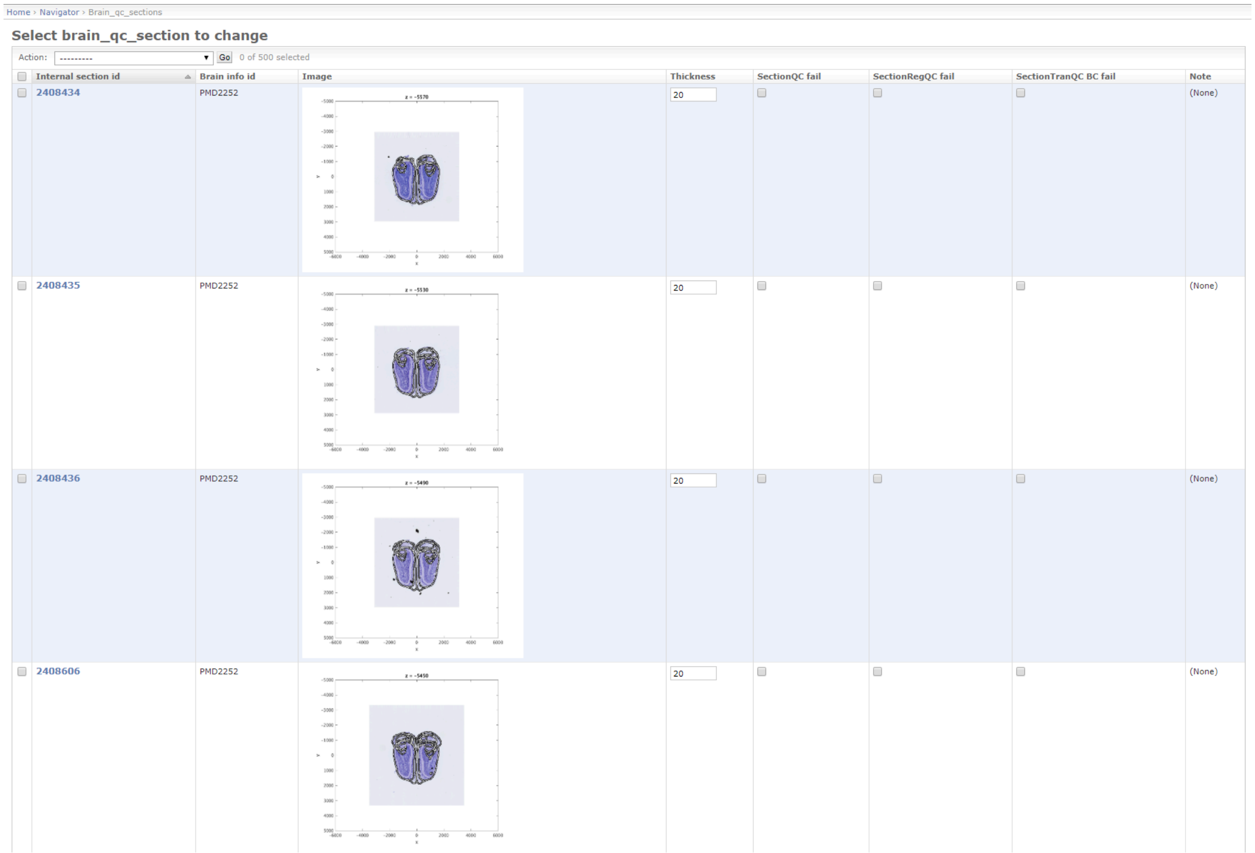

Figure S1**:** Web based quality control interface allows users to view registered sections with atlas annotation overlays and verify imaging parameters before the expensive process of transforming high resolution images begins.

Accurate rigid alignment between Nissl sections and sections of other modalities is critical, and these mappings occasionally fail due to findinging local minima in a nonconvex optimization problem. We have created an interactive tool where a user chooses corresponding landmark points in pairs of images. A rigid transformation is displaced and updated with each new landmark. This manual alignment can either be used directly, or treated as an initial guess for the automatic rigid alignment optimization procedure.

Transformation: The high resolution images can be transformed after the registered brain passes QC or is manually corrected. Due to the quality of the tape transfer method, only rigid registration is applied to each 2D brain section, which can be applied very quickly using matrix arithmetic, as opposed to interpolating a displacement vector field. Each section is transformed and padded to a fixed image size of 24000 * 24000 pixels. The 10um atlas is deformed nonlinearly and upsampled to image resolution. In

order to display the image and annotation on the viewer, brain sections are compressed into JP2 format, and annotation is converted into geojson.

Running time: Registration is run on a shared supercomputer cluster at Cold Spring Harbor Laboratory (CSHL). Since it is a shared resource, the number of jobs that can run at the same time varies. We require about 16 CPU threads and 24G memory for one brain to finish in 8 hours. Compute time varies considerably depending on data and parameters chosen however, and the serial section alignment example we include in our github repository takes only about 12 minutes to complete (for 668 Nissl slices). Typically, 30 brains can be registered in parallel. Applying the transformation to high resolution images requires relatively high memory, and therefore is run on a multi-node custom built cluster. The cluster has 8 nodes and each node has 72 CPU threads, 188G memory and 2 Nvidia GTX 2080TI GPUs. Each node can transform one brain in 2.5 hours, so the maximum capacity is about 80 brains per day.

Supplementary Note 2: Diffeomorphometry

Diffeomorphometry supplement

Supplementary note for Tward, et al. 2025

Introduction

Understanding typicality and variability in brain anatomy as a quantitative science has been the goal of the Computational Anatomy program[1]. Several important mathematical and algorithmic results have been established, which we draw upon for accurate brain registration and statistical analysis. The key feature of this field is to model observed anatomical images as spatial deformations of a well characterized template. Employing the deformable template approach allows these transformations, known as diffeomorphisms, to become the objects of study when describing shape, rather than images themselves. The quantitative study of shape using diffeomorphisms has become known as diffeomorphometry [2]. In this supplement we review some of these important details.

Diffeomorphisms and their action on images

Diffeomorphic transformations are differentiable invertible coordinate mappings with a differentiable inverse. Given a background space X (typically a subset of $\mathbb{R}^2$ or $\mathbb{R}^3$ real numbers), we denote them by $\varphi : X \to X$, which maps $x \mapsto \varphi(x)$. The set of diffeomorphisms form a mathematical group under the composition operation ($[\varphi_A \circ \varphi_B](x) = \varphi_A(\varphi_B(x))$), with identity elements, existence of inverse, and associativity (but not commutativity). This set is not a vector space, meaning linear combinations of diffeomorphisms does not generally lead to a diffeomorphism, making standard linear modeling techniques inappropriate.

Given an anatomical image $I : X \to \mathbb{R}$, diffeomorphisms deform them via composition with the inverse $\varphi \cdot I = I \circ \varphi^{-1}$. While sometimes counterintuitive, this left action obeys necessary associativity properties $\varphi_B \cdot (\varphi_A \cdot I) = (\varphi_B \circ \varphi_A) \cdot I$. It is immediately clear that working with invertible transformations is essential to transform points ($x_i \mapsto \varphi(x_i)$) and images ($I \mapsto I \circ \varphi^{-1}$) consistently.

Derivatives define scale change

Because φ is a diffeomorphism, its Jacobian matrix $D\varphi$ is well defined and invertible everywhere in space. This matrix defines how a small cube in the atlas is transformed into a small paralelipiped in the observed volume. The ratio of the paralellipiped's volume to that of the cube is determined by the determinant of the Jacobian. Working with the cube root changes units from volume to length, and we call the result the scale

change. Studying this object as a means to quantify local size and shape differences has been well established [3].

Generating diffeomorphisms via smooth flows

In computational anatomy diffeomorphisms are generated by integrating time varying velocity fields, which can be thought of colloquially as “displacement equals velocity times time”. We denote a time varying velocity field $v_t : X \to \mathbb{R}^D$ where D is typically 2 or 3. A diffeomorphism is constructed by integrating Euler’s equation from time 0 to 1

$$\frac{d}{dt}\varphi_t = v_t(\varphi_t), \text{ with } \varphi_0 = \text{identity} \; . \qquad (1)$$

The inverse can be constructed from the optical flow equation:

$$\frac{d}{dt}\varphi_t^{-1} = -D\varphi_t^{-1} v_t, \text{ with } \varphi_0^{-1} = \text{identity} \; . \qquad (2)$$

In image registration we work with the inverse equation which is solved numerically using Semi Lagrangian advection[4]. In our notation, when no time index is specified on φ_t time $t = 1$ is implied.

The vector space of smooth flows

To guarantee that integrating a velocity field leads to a diffeomorphism, it must have a sufficient number of continuous derivatives [5]. This is achieved in practice by modeling v_t as belonging to a Reproducing Hilbert Space of smooth functions V, with an inner product that penalizes high frequency components:

$$\langle u, v \rangle_V = \int [Lu]^T(x)[Lv](x)dx, \quad L = (\text{identity} - \alpha^2 \text{Laplacian})^2 \qquad (3)$$

,

where α is a parameter with units of length that controls the length scale of smoothness. This leads to an associated norm which allows us to speak of length and distance in the space of diffeomorphisms, $\|v\|_V^2 = \langle v, v \rangle_V$.

Registration as an optimal control problem

Image registration can be posed as an optimization problem that balances accuracy in a least squares sense with smoothness in terms of the norm defined above. An atlas image I can be transformed to a target image J by solving the optimization problem:

$$v^* = \arg\max_v \frac{1}{2\sigma_R^2}\int_0^1 \|v_t\|_V^2 dt + \frac{1}{2\sigma_M^2}\int |I(\varphi_1^{-1}(x)) - J(x)|^2 dx \ . \qquad (4)$$

For this problem, α, σ_R^2, and σ_M^2 are user specified constants that define the balance between smoothness scale, smoothness strength, and matching accuracy (respectively). This problem was originally solved in [6], and has been approached by various other researchers (and ourselves) with different accuracy functionals.

Optimal trajectories are geodesics

An important property of optimal solutions is that v_t^* defines a length minimizing curve between identity and φ_1. These geodesic curves are characterized by a conservation of momentum equation [7]. Let $m = L^*Lv$. Here $*$ refers to the adjoint of the linear operator, which is defined implicitly by

$$\int [Lu]^T(x)[Lv](x)dx = \int [u]^T(x)[L^*Lv](x)dx \ . \qquad (5)$$

Note that the operator L defined above is self adjoint ($L^* = L$). The geodesic curves are characterized by

$$\frac{d}{dt}m + Dmv + m\mathrm{div}v + Dv^T m = 0 \ . \qquad (6)$$

Therefore, given a velocity vector field defined at $t = 0$, one can reconstruct the entire optimal trajectory v_t and thus the diffeomorphism φ_1. Since v_0 lies in the vector space V, we use v_0 as a linear parameterization of φ_1. In the language of if differential geometry, v_0 is a vector in the tangent space to the diffeomorphism group at the identity element.

Tangent space kernel PCA

Because v_0 lies in a linear space, it can be modeled (when sampled on a voxel grid) as a multivariate Gaussian. In this setting we can use principal component analysis to compute uncorrelated, orthogonal modes of variability. Because we have defined v_0 as belonging to the vector space V, orthogonal must be interpreted as $\langle u, v\rangle_V = 0$. This procedure is described in detail in [8], and we summarize it here.

We let N be the number of voxels in our atlas image, and M be the number of samples of v_0 we have measured. Let X be a $3N \times M$ data matrix formed by vectorizing and stacking each v_0, and X_0 be centered by subtracting the mean at each voxel $\bar{X}$. Instead of working with the covariance matrix directly, we compute the Gram matrix of inner products as $G = X_0^T K X_0$ where K is the $3N \times 3N$ kernel matrix that applies L^*L to our vectorized velocity fields. In practice K is applied using Fourier transforms, and not stored as a matrix. We then compute the eigendecomposition $G = VDV^T$, for

V unitary and D diagonal. The variance of the i-th orthogonal mode is given by $\sigma_i^2 = D_{ii}/N$, and the modes themselves are given by the columns of $U = X_0 V D^{-\frac{1}{2}}$, which are orthogonal with respect to our inner product.

For the i-th mode of variation, we can initialize Eq (6) with $v_0 = \bar{X} + s\sigma_i U_{\cdot i}$ and reconstruct a diffeomorphism φ_1 that is s standard deviations from the mean in the direction of the first mode. From this diffeomorphism we can analyze scale change as described above.

References

1. Grenander, U. & Miller, M. I. Computational anatomy: An emerging discipline. *Quart. Appl. Math.* **56**, 617–694 (1998).
2. Miller, M. I., Younes, L. & Trouvé, A. Diffeomorphometry and geodesic positioning systems for human anatomy. *Technology* **2**, 36 (2014).
3. Ashburner, J. & Friston, K. J. Voxel-based morphometry—the methods. *Neuroimage* **11**, 805–821 (2000).
4. Staniforth, A. & Côté, J. Semi-Lagrangian Integration Schemes for Atmospheric Models—A Review. *Mon. Weather Rev.* **119**, 2206–2223 (1991).
5. Dupuis, P., Grenander, U. & Miller, M. I. VARIATIONAL PROBLEMS ON FLOWS OF DIFFEOMORPHISMS FOR IMAGE MATCHING. *Quart. Appl. Math.* **56**, 587–600 (1998).
6. Beg, M. F., Miller, M. I., Trouvé, A. & Younes, L. Computing Large Deformation Metric Mappings via Geodesic Flows of Diffeomorphisms. *Int. J. Comput. Vis.* **61**, 139–157 (2005).
7. Miller, M. I., Trouvé, A. & Younes, L. Geodesic Shooting for Computational Anatomy. *J. Math. Imaging Vis.* **24**, 209–228 (2006).
8. Vaillant, M., Miller, M. I., Younes, L. & Trouvé, A. Statistics on diffeomorphisms via tangent

space representations. *Neuroimage* **23 Suppl 1**, S161–9 (2004).

Supplementary Note 3: Stereology

Sterology correction factor

Supplementary note for Tward, et al. 2025

Introduction

In this supplement we summarize how we derived our stereological correction factor for cell density analysis.

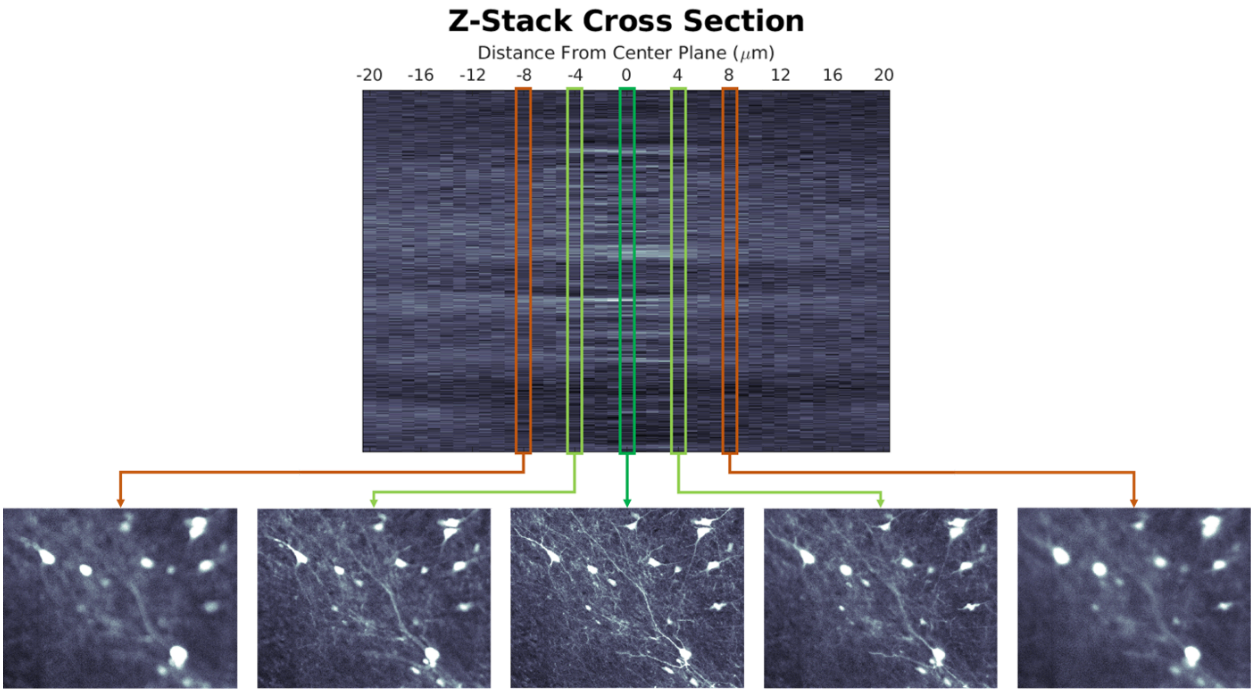

Figure S2. Top panel: a cross section of a window in a z-stack obtained using the same imaging system as analyzed datasets. Note the bright stripes of signal; each corresponds to a neuron or cluster of adjacent neurons. Bottom panel: Example images of the window in the z-stack at the center plane of the section and also at 4μm and 8μm from the center plane in both directions. Green boxes indicate all cell bodies and processes are still in focus (at 4μm) while orange boxes (at 8μm) indicate loss of focus leading to loss of defined cell bodies or processes.

Stereological correction procedure

We calculate an effective post-processing section thickness and attendant cell density estimation correction factor based on a z-stack obtained on the same imaging system as the datasets analyzed in the present manuscript (Nanozoomer HT 2.0). The top panel Figure S2 shows a cross-section of a window through the z-stack of section cut

with the microtome set for 20μm section thickness, with distance from the center plane in the z-direction given in μm. The estimation procedure is detailed below:

1. We let the original section thickness be T (which is set during microtomy to 20μm).
   a. Note that cutting on the microtome is subjected to some variation (1-2 μm) around the nominal mean value of 20μm. Cutting will produce both slightly thicker and thinner sections. To a first approximation we therefore adopt a constant section thickness of 20μm
2. Let the measured in-plane density (from running cell detection) be ρ_{2D}. We need to know the relation between ρ_{2D} and ρ_{3D}, where the latter is the 3D volumetric density. The relation between the two may be written as:
   a. $\rho_{3D} = \rho_{2D} / t$, where t = an effective section thickness for converting the 2D densities to 3D densities.
3. The data shows that the histological processing shrinks the physical thickness of the section. We estimate the effective post-processing section thickness, to be 10-12μm as follows:
   a. In example images (bottom panel of Figure S2) of the focal plane at 4μm from the center plane in either direction, we note clear neuronal cell bodies and processes in focus. By 8μm from the center plane, the imaged z-plane is out of focus; however, cell bodies are still discernible, suggesting this z-plane is outside the edge of the tissue in both directions.
   b. In the top panel of Figure S2 showing the z-direction cross section, we note that the brightest fluorescent signals (each one indicates a cell or group of adjacent cells) go to ~5-6μm from the center plane, lending support to a post processing section thickness between 10-12μm.
4. Based on these observations we assume that if we detect every object in the optical section, small and large, we will capture any cell that is wholly or partially in that section. With this assumption (which we justify based on the z-stack data above), the effective thickness of a slab over which the observed cell centers are distributed, is given by t=T+2R, where R is the radius of the cell nuclei (which we assume to be spheres for this purpose; note that the in-plane profiles we observe are largely circular).
5. Assuming that the cell centers follow a spatial Poisson distribution with a density parameter of ρ_{3D}, we then have the following equation:
   a. $\rho_{2D} = \rho_{3D} \times (T+2d)$, or $\rho_{3D} = \rho_{2D} / (T+2R)$

Setting T=20μm and R=5μm we have an effective thickness of 30μm. We use this conversion factor to compute the 3D densities from the 2D densities in the original image data (before any diffeomorphic mapping).

Supplementary Note 4: Registration refinement

Mapping refinement

Supplementary note for Tward, et al. 2025

Introduction

Quality control and mapping accuracy is key for scientific insight. While annotations that result from atlas mapping can be updated by expert anatomists, our analysis relies on a consistency between annotations and mappings that are also studied. We therefore refine mappings based on manual annotations as opposed to updated annotations directly.

The manual annotation cost function

If the result of a mapping is not satisfactory, users may annotate observed images J^i with one or more binary masks M^i, each corresponding to some set of labels in Allen Reference Atlas. A synthesized version $\hat{M}^i$ is generated from the Allen atlas, which is expected to match only in the region indicated (i.e. the user need only perform segmentations in regions with inaccuracies, not everywhere in the image).

$$\arg\min_{\varphi^0, A, \varphi^i, R^i, f^i} \mathrm{Reg}^0(\varphi^0) + \sum_i \mathrm{Reg}^i(\varphi^i) + \frac{1}{2\sigma^2}\int |\hat{J}^i(x) - J^i(x)|^2 \pi^i(x) dx + \frac{1}{2\sigma_M^2}\int |\hat{M}^i(x) - M^i(x)|^2 M^i(x) dx \ . \qquad (1)$$

This minimization problem is initialized using the previous solution, which is typically close to optimal, reducing computation time needed.

Supplementary Note 5: Validation

Mapping validation

Supplementary note for Tward, et al. 2025

Introduction

Here we detail the experiments used to validate our registration pipeline using simulated images.

Part 1: Validation using simulated images

We evaluated the capabilities of our novel loss function, as compared to several alternatives.

1. Contrast matching in blocks for color images (our proposed method)
2. Contrast matching in blocks for grayscale images
3. Global contrast matching for color images
4. Global contrast matching for grayscale images
5. Normalized cross correlation (NCC) for grayscale images
6. Mutual information (MI) for grayscale images.

Normalized cross correlation is commonly used in ANTs, and mutual information is used in many registration approaches. One important advantage of our proposed loss function is that it accommodates color images, but for a “more fair” comparison with the grayscale image methods NCC and MI, we also considered grayscale images by using the first principal component of our color image.

To make efficient use of existing atlas annotations, we conducted a simulation study by generating 2D images. Starting with atlas labels we assigned random colors to each section, with correlations among gray matter structures, and among white matter structures. The cell-poor molecular layer of the cortex was also correlated with the intensity of white matter structures. We applied random affine transforms and diffeomorphisms to an atlas and target image, and applied missing tissue, streak artifacts, and inhomogeneity to a target image. Some examples are shown below.

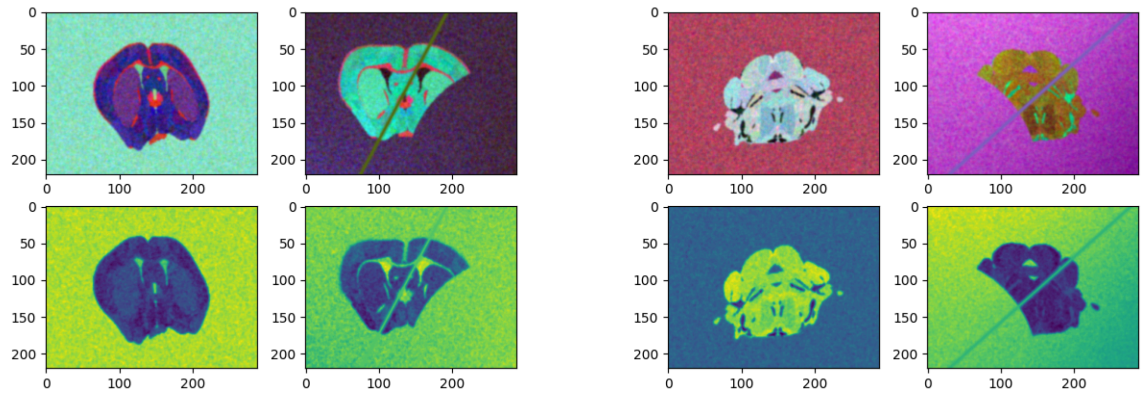

Figure S3. Top row shows simulated color images, and bottom row shows their conversion to grayscale using the first principal component. For each slice the left side shows our atlas (or moving) image, and the right side shows our target (or fixed) image.

We computed alignments from atlas to target using exactly the same deformation model in all cases, but with different loss functions. Regularization terms and gradient descent step sizes were tuned in each case to provide good results, and each experiment used 1500 iterations of gradient descent (enough to converge). The experiment was repeated for 43 coronal slices, 250 microns apart, at 25 micron resolution, spanning the anterior-posterior extent of the brain.

For each labeled anatomical structure, we computed the Dice overlap score (volume of intersection divided by average volume) between the transformed atlas and target labels. We compare our method to alternatives by presenting these scores as scatter plots, showing mean and standard error of the mean for each structure. In the plots below, the identity line is shown in black at 45 degrees, and when points appear toward the lower right, our proposed method (x axis) is outperforming the alternative (y axis).

First we show that our proposed method (contrast matching in blocks) strongly outperforms our previous work (global contrast matching) for both color and grayscale images.

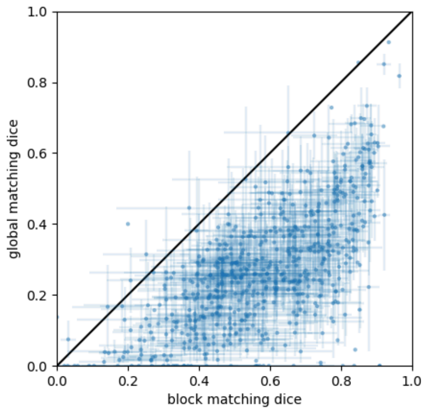

Figure S4. Scatter plot showing dice overlap after registration for our proposed method (x axis), and our earlier method which did not do local contrast matching in blocks (y axis).

Second we show that our proposed method strongly outperforms mutual information, both for color and grayscale images. In fact, global methods including MI and global contrast matching often fail in the presence of inhomogeneity.

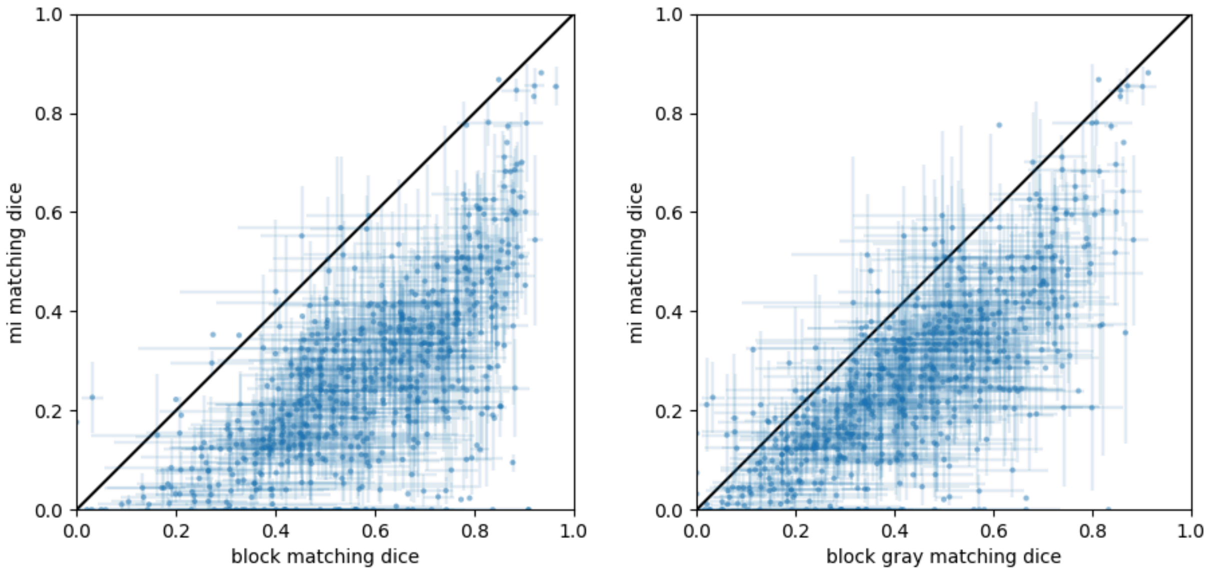

Figure S5. Scatter plot showing dice overlap after registration for our proposed method (x axis), and for mutual information (y axis). On the left our method operates on color images, whereas on the right it operates on grayscale.

Third we show that our proposed method strongly outperforms NCC when using color images, and also outperforms NCC when using grayscale images.

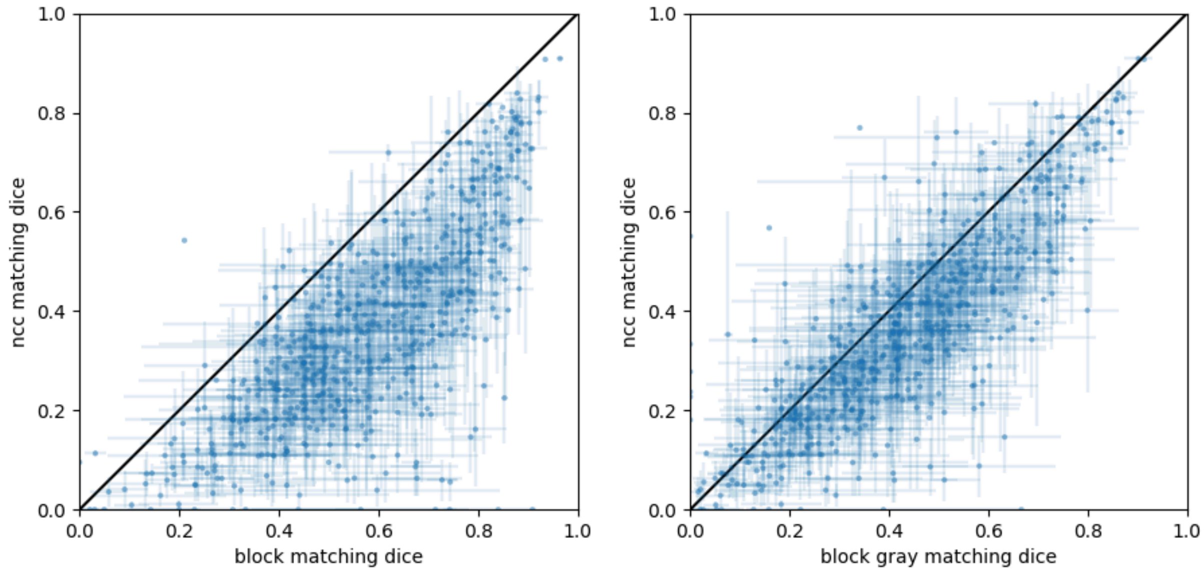

Figure S6. Scatter plot showing dice overlap after registration for our proposed method (x axis), and for normalized local cross correlation (y axis). On the left our method operates on color images, whereas on the right it operates on grayscale.

Because NCC only models linear transformations of contrast, it often performs poorly in regions where the contrast relationship between atlas and target is nonlinear. This often occurs when registering brightfield nissl images to brightfield myelin in the molecular layer. In a nissl stain, the contrast profile moves from light (background), to medium (molecular layer), to dark (gray matter). In a myelin stain, the contrast profile moves from light (background), to dark (molecular layer), to medium (gray matter). Below we show registration results using a pair of images generated to appear “nissl like” and “myelin like”. We show the overlap of brain masks between transformed atlas and target, revealing that our nonlinear contrast transform approach successfully aligns the molecular layer, but NCC does not.

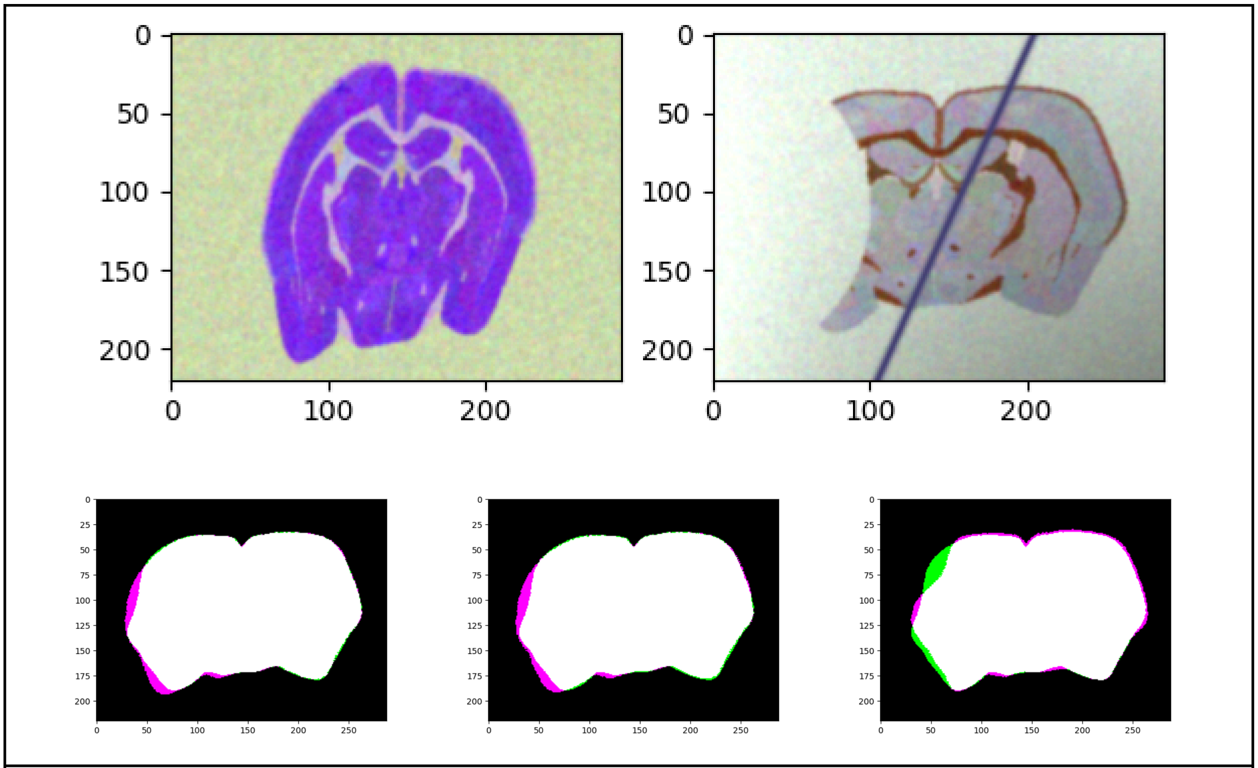

Figure S7. Top left: “nissl-like” image, top right: “myelin-like” image. Bottom row shows transformed atlas brain map (magenta) and target brain (green). Where they overlap the color is white. The left side shows results for our method with color images, the center shows results of our method with a grayscale image, and the right shows the results of the local cross correlation method. In the latter case, the magenta layer near the top and right side of the brain indicates poor performance near the molecular layer.

We also highlight molecular layer structures on a scatterplot of Dice scores below.

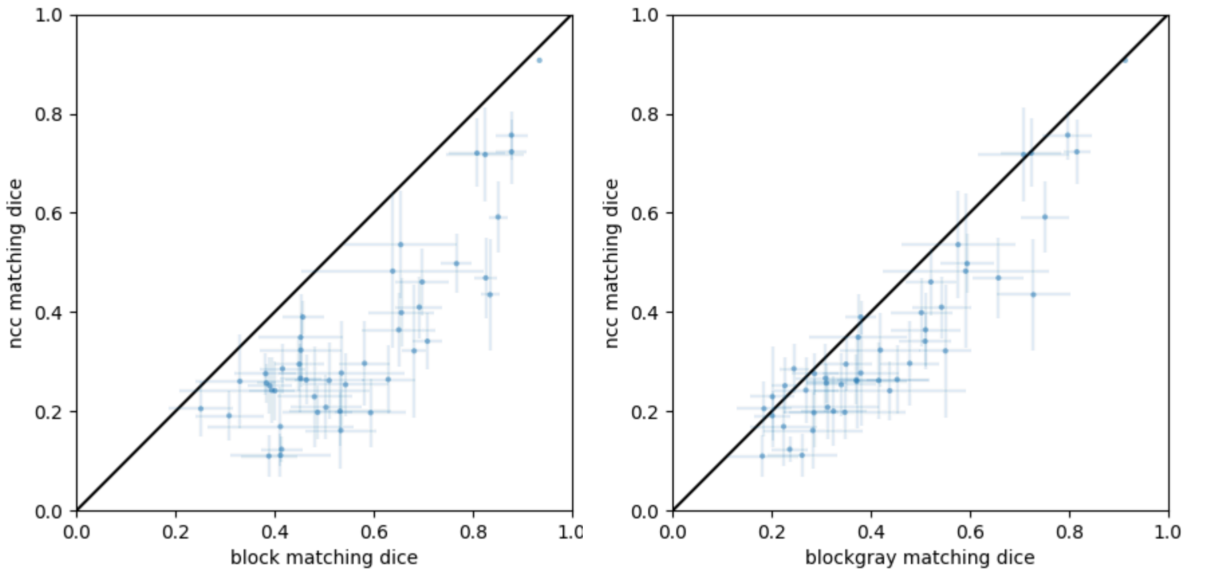

Figure S8. Scatter plot showing dice overlap after registration for our proposed method (x axis), and for mutual normalized cross correlation (y axis), for molecular layer structures only. On the left our method operates on color images, whereas on the right it operates on grayscale.

In all the above experiments we jointly optimized over affine and deformation parameters, but we used a known affine transform as an initialization. To understand the sensitivity to initial conditions, we repeated the experiment with the affine transform initialized to identity. In this case dice overlap scores for our method were generally lower, reduced by a median across all structures of 16.0%. As a comparison, for NCC, Dice overlap scores were reduced by a median of 22.4% (from an already lower baseline).

Part 2: Comparison to Chandrashekhar 2021

Here we justify our specific claims made comparing to our previous work (Chandrashankar 2021), which computes contrast transformations through spatially varying polynomial coefficients, whose smoothness is regularized using the same approach as velocity fields are regularized in LDDMM.

“The blockwise approach we developed here tends to produce more accurate results”,

Dice overlap scores for our proposed method, and for Chandrashekhar 2021, are shown below. We note that our method outperforms the alternative for 74.4% of brain structures, with an average Dice increase of 0.087.

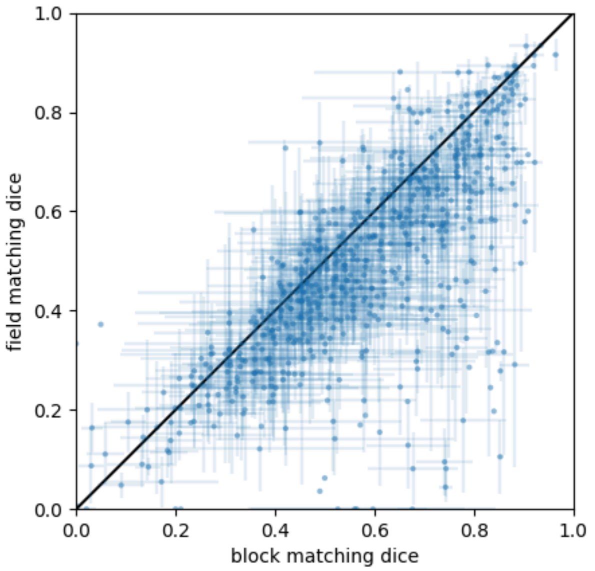

Figure S9. Scatter plot showing dice overlap after registration for our proposed method (x axis), and for the Chandrashekhar 2021 method (y axis).

“converges more quickly during optimization”

Here we show example results using the “nissl-like” and “myelin-like” images presented above. First we show results for our proposed approach:

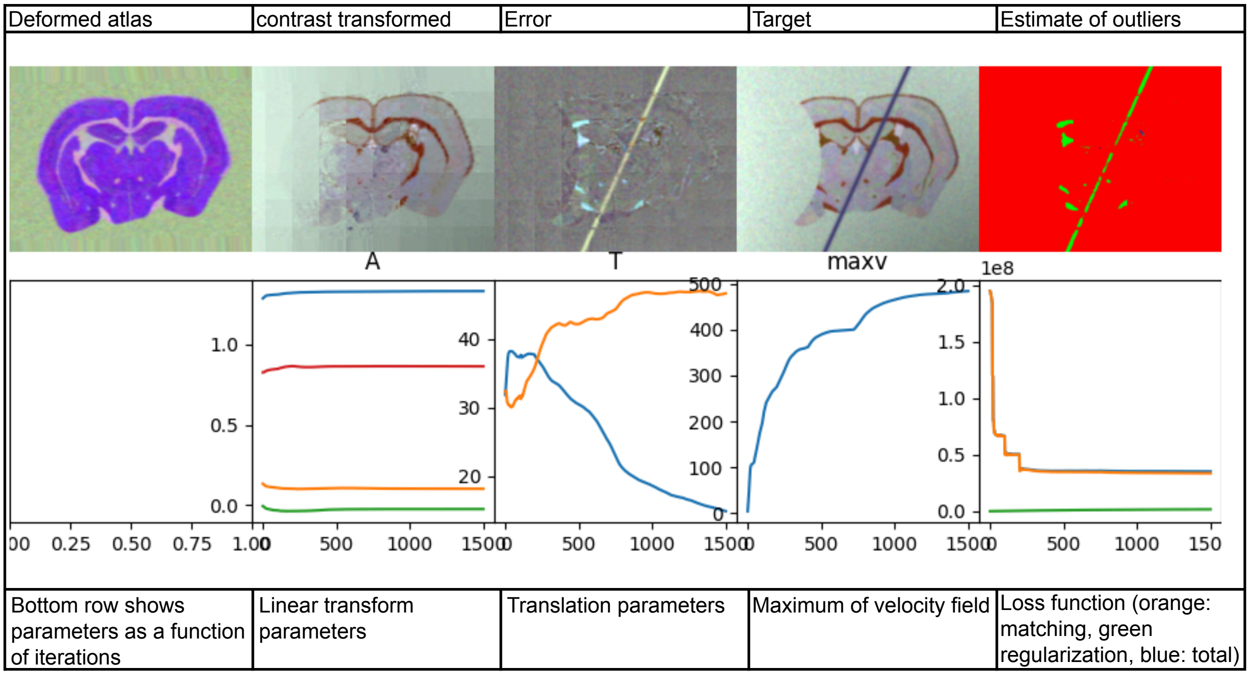

Figure S10.

Second we show results for the Chandrashekhar approach. Note that the value of the loss function has not converged after 1500 iterations of gradient descent.

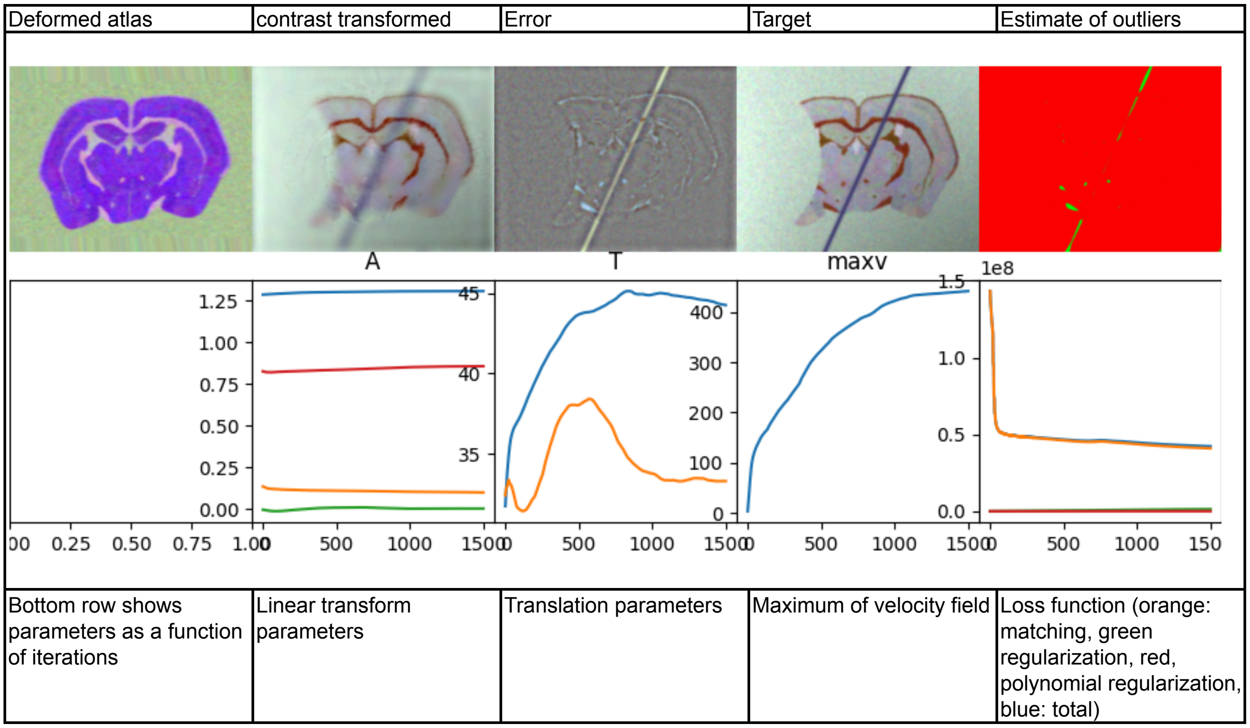

Figure S11.

The computation time for this example was 49 seconds for our proposed method, and 93 seconds for the Chandrashekhar approach. The time differences are owed largely to

the fact that the coefficients of regularized polynomial fields cannot be computed in closed form, whereas polynomials in blocks can.

“parallelizes more efficiently”

We make this claim as pytorch has built in tools to parallelize over a leading batch dimension for each tensor, where parallelization can be done on one or multiple gpus, or cores of a cpu. In our approach each block corresponds to a batch dimension. In the polynomial fields work no such parallelization is possible (although the technique does benefit from the efficiency of a Fast Fourier Transform).

“and requires fewer parameters for users to select.”

In the approach we present a user only needs to select a block size (usually 1 number, but potentially 3 numbers for blocks that are not cubes).

In our earlier work a user needs to specify:

1. A length scale for smoothness regularization (usually 1 number, but potentially 3)
2. A power of Laplacian for smoothness regularization
3. An overall magnitude for smoothness regularization
4. A gradient descent stepsize
5. A number of iterations to update the contrast transform each time we update the registration parameters (in our implementation we had used 30 the first iteration, and 5 thereafter)

Part 3: Overfitting with blockwize contrast transforms

Below we show an example where the block size is chosen too large (256 pixels). Notice that inhomogeneity is not modeled well, many pixels are flagged as outliers, and the accuracy is low.

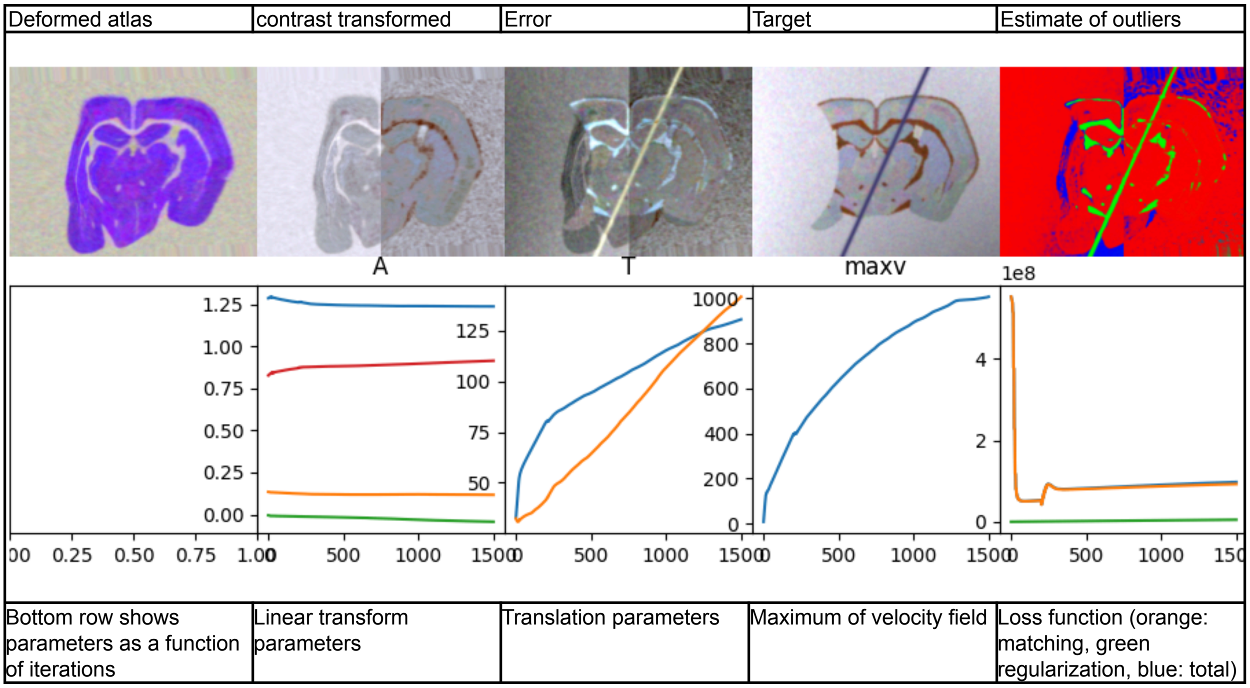

Figure S12.

Below we show an example where the block size is chosen too small (8 pixels). Notice the atlas image is deformed very little and does not match the shape of the target, but its contrast is transformed to match the target almost exactly.

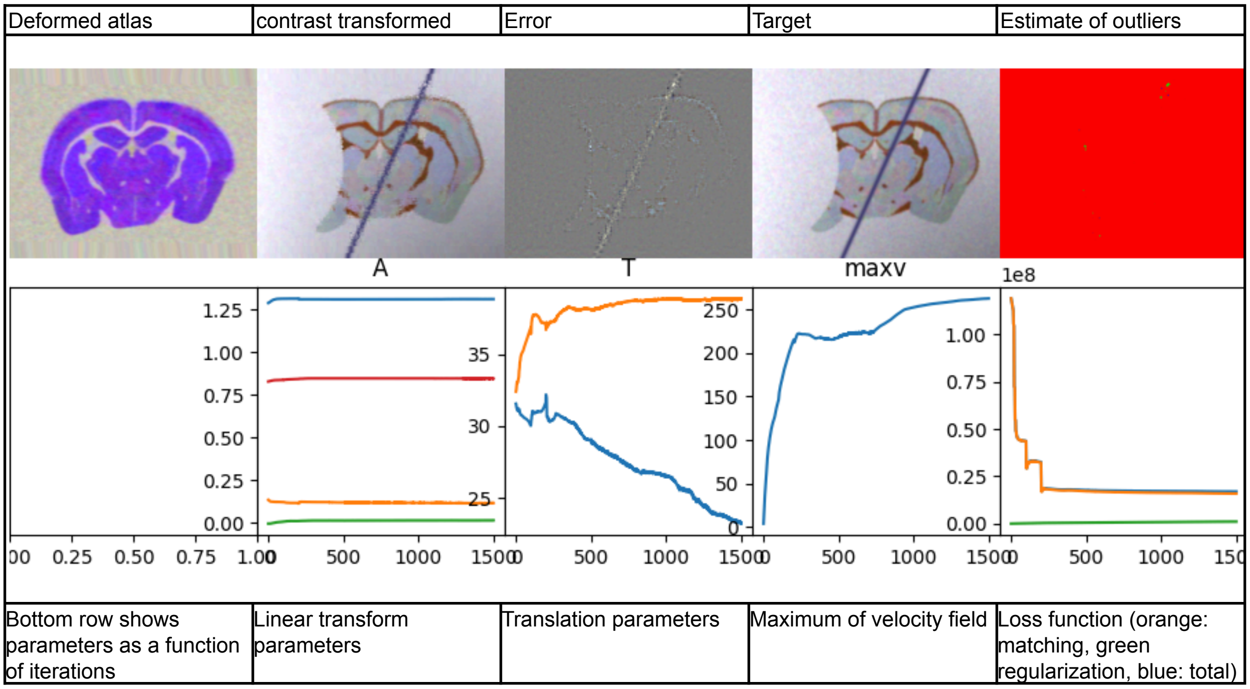

Figure S13.

These should be contrasted with a block size of 32 shown in Part 2 above, which finds a good balance between these two extremes and leads to good quality results.

References

Chandrashekhar V, Tward DJ, Crowley D, Crow AK, Wright MA, Hsueh BY, Gore F, Machado TA, Branch A, Rosenblum JS, Deisseroth K. CloudReg: automatic terabyte-scale cross-modal brain volume registration. Nature methods. 2021 Aug;18(8):845-6.

Supplementary Note 6: Scale change

Tables of scale changes

Supplementary note for Tward, et al. 2025

1 Male and female aggregated

Table S1: The relative growth corresponding to 7 weeks is quantified for all structures at level 5 of the Allen atlas ontology. They are sorted by magnitude of log scale change and grouped by sign. Scale change is relative to spatially uniform background.

structure	sign	mean scale change	mean displacement (μm)
cerebellar commissure	1	1.06	45
doral tegmental decussation	1	1.05	57
medial longitudinal fascicle	1	1.05	87
Fastigial nucleus	1	1.04	70
crossed tectospinal pathway	1	1.04	46
internal capsule	1	1.04	39
cerebal peduncle	1	1.04	38
nigrostriatal tract	1	1.04	46
corticospinal tract	1	1.04	27
motor root of the trigeminal nerve	1	1.04	39
fiber tracts	1	1.04	40
middle cerebellar peduncle	1	1.04	37
lateral ventricle	1	1.04	44
arbor vitae	1	1.04	46
cervicothalamic tract	1	1.03	51
oculomotor nerve	1	1.03	40
subependymal zone	1	1.03	51
stria terminalis	1	1.03	41
choroid plexus	1	1.03	56
auditory radiation	1	1.03	40
optic tract	1	1.03	52
facial nerve	1	1.03	76
corpus callosum body	1	1.03	45
external medullary lamina of the thalamus	1	1.03	33
cochlear nerve	1	1.03	46
Medulla	1	1.03	61
Vermal regions	1	1.03	57
inferior cerebellar peduncle	1	1.03	40
ventral tegmental decussation	1	1.02	70
fornix system	1	1.02	32
superior cerebelar peduncles	1	1.02	37
optic nerve	1	1.02	29
genu of corpus callosum	1	1.02	46
trochlear nerve	1	1.02	52
optic radiation	1	1.02	32
Pons	1	1.02	45
mammillary related	1	1.02	60
Midbrain behavioral state related	1	1.02	47
Midbrain motor related	1	1.02	59
cerebral aqueduct	1	1.02	98
Midbrain	1	1.02	57

posterior commissure	1	1.02	46
Interposed nucleus	1	1.02	40
Cerebellum	1	1.02	45
supra-callosal cerebral white matter	1	1.02	26
vomeronasal nerve	1	1.02	38
Dentate nucleus	1	1.02	50
third ventricle	1	1.02	38
vestibular nerve	1	1.02	32
cingulum bundle	1	1.02	38
pyramid	1	1.02	33
supraoptic commissures	1	1.02	37
genu of the facial nerve	1	1.02	92
rubrospinal tract	1	1.02	43
sensory root of the trigeminal nerve	1	1.02	37
Hypothalamus	1	1.02	40
Vestibulocerebellar nucleus	1	1.02	74
Pallidum	1	1.02	42
Hemispheric regions	1	1.02	44
corpus callosum anterior forceps	1	1.02	28
corpus callosum splenium	1	1.02	23
lateral recess	1	1.01	57
Striatum	1	1.01	37
anterior commissure olfactory limb lateral	1	1.01	39
olfactory tract general	1	1.01	27
optic chiasm	1	1.01	20
corpus callosum posterior forceps brachium	1	1.01	22
of the superior colliculus epithalamus	1	1.01	40
related	1	1.01	52
superior colliculus commissure	1	1.01	54
pyramidal decussation	1	1.00	32
solitary tract	-1	0.93	48
central canal spinal cord/medulla	-1	0.94	65
fourth ventricle	-1	0.97	73
Thalamus	-1	0.97	51
corpus callosum extreme capsule	-1	0.98	16
amygdalar capsule	-1	0.98	20
Midbrain sensory related	-1	0.98	44
Cortical plate	-1	0.98	33
Cortical subplate	-1	0.99	32
anterior commissure temporal limb	-1	0.99	40
olfactory nerve layer of main olfactory bulb	-1	0.99	24
medial forebrain bundle	-1	0.99	70
solitary tract	-1	0.93	48
central canal spinal cord/medulla	-1	0.94	65

Table S2: The relative growth corresponding to 7 weeks is quantified for cortical structures at level 7 of the Allen atlas ontology. They are sorted by magnitude of log scale change and grouped by sign. Scale change is relative to spatially uniform background.

structure	sign	mean scale change	mean displacement (μm)
Hippocampal region	1	1.01	40
Anterior olfactory nucleus	1	1.01	40
Main olfactory bulb	1	1.01	29
Postpiriform transition area	1	1.01	42
Piriform-amygdalar area	1	1.00	33
Retrosplenial area	-1	0.96	56
Posterior parietal association areas	-1	0.97	43
Infralimbic area	-1	0.97	40
Anterior cingulate area	-1	0.97	66
Visual areas	-1	0.97	38
Dorsal peduncular area	-1	0.97	48
Taenia tecta	-1	0.98	57
Temporal association areas	-1	0.98	24
Somatosensory areas	-1	0.98	25
Auditory areas	-1	0.98	25
Visceral area	-1	0.98	23
Ectorhinal area	-1	0.98	16
Gustatory areas	-1	0.98	20
Agranular insular area	-1	0.98	21
Orbital area	-1	0.98	27
Somatomotor areas	-1	0.98	29
Accessory olfactory bulb	-1	0.98	34
Prelimbic area	-1	0.99	33
Perirhinal area	-1	0.99	15
Hippocampal formation	-1	0.99	39
Olfactory areas	-1	0.99	29
Retrohippocampal region	-1	0.99	35
Piriform area	-1	0.99	25
Frontal pole cerebral cortex	-1	0.99	31
Cortical amygdalar area	-1	0.99	38
Nucleus of the lateral olfactory tract	-1	1.00	32
Retrosplenial area	-1	0.96	56
Posterior parietal association areas	-1	0.97	43
Infralimbic area	-1	0.97	40
Anterior cingulate area	-1	0.97	66
Visual areas	-1	0.97	38
Dorsal peduncular area	-1	0.97	48
Taenia tecta	-1	0.98	57
Temporal association areas	-1	0.98	24
Somatosensory areas	-1	0.98	25
Auditory areas	-1	0.98	25
Visceral area	-1	0.98	23
Ectorhinal area	-1	0.98	16
Gustatory areas	-1	0.98	20
Agranular insular area	-1	0.98	21
Orbital area	-1	0.98	27
Somatomotor areas	-1	0.98	29
Accessory olfactory bulb	-1	0.98	34
Prelimbic area	-1	0.99	33

Table S3: The pattern of correlations and anticorrelations in the largest mode of variation is quantified for all structures at level 5 of the Allen atlas ontology. They are sorted by magnitude of log scale change and grouped by sign. Structures with the same sign are correlated with each other, and anticorrelated with those of opposite sign.

structure	sign	mean scale change	mean displacement (μm)
cerebellar commissure	1	1.06	45
doral tegmental decussation	1	1.05	57
medial longitudinal fascicle	1	1.05	87
Fastigial nucleus	1	1.04	70
crossed tectospinal pathway	1	1.04	46
internal capsule	1	1.04	39
cerebal peduncle	1	1.04	38
nigrostriatal tract	1	1.04	46
corticospinal tract	1	1.04	27
motor root of the trigeminal nerve	1	1.04	39
fiber tracts	1	1.04	40
middle cerebellar peduncle	1	1.04	37
lateral ventricle	1	1.04	44
arbor vitae	1	1.04	46
cervicothalamic tract	1	1.03	51
oculomotor nerve	1	1.03	40
subependymal zone	1	1.03	51
stria terminalis	1	1.03	41
choroid plexus	1	1.03	56
auditory radiation	1	1.03	40
optic tract	1	1.03	52
facial nerve	1	1.03	76
corpus callosum body	1	1.03	45
external medullary lamina of the thalamus	1	1.03	33
cochlear nerve	1	1.03	46
Medulla	1	1.03	61
Vermal regions	1	1.03	57
inferior cerebellar peduncle	1	1.03	40
ventral tegmental decussation	1	1.02	70
fornix system	1	1.02	32
superior cerebelar peduncles	1	1.02	37
optic nerve	1	1.02	29
genu of corpus callosum	1	1.02	46
trochlear nerve	1	1.02	52
optic radiation	1	1.02	32
Pons	1	1.02	45
mammillary related	1	1.02	60
Midbrain behavioral state related	1	1.02	47
Midbrain motor related	1	1.02	59
cerebral aqueduct	1	1.02	98
Midbrain	1	1.02	57
posterior commissure	1	1.02	46
Interposed nucleus	1	1.02	40
Cerebellum	1	1.02	45
supra-callosal cerebral white matter	1	1.02	26
vomeronasal nerve	1	1.02	38
Dentate nucleus	1	1.02	50
third ventricle	1	1.02	38
vestibular nerve	1	1.02	32
cingulum bundle	1	1.02	38
pyramid	1	1.02	33
supraoptic commissures	1	1.02	37

genu of the facial nerve	1	1.02	92
rubrospinal tract	1	1.02	43
sensory root of the trigeminal nerve	1	1.02	37
Hypothalamus	1	1.02	40
Vestibulocerebellar nucleus	1	1.02	74
Pallidum	1	1.02	42
Hemispheric regions	1	1.02	44
corpus callosum anterior forceps	1	1.02	28
corpus callosum splenium	1	1.02	23
lateral recess	1	1.01	57
Striatum	1	1.01	37
anterior commissure olfactory limb	1	1.01	39
lateral olfactory tract general	1	1.01	27
optic chiasm	1	1.01	20
corpus callosum posterior forceps	1	1.01	22
brachium of the superior colliculus	1	1.01	40
epithalamus related	1	1.01	52
superior colliculus commissure	1	1.01	54
pyramidal decussation	1	1.00	32
solitary tract	-1	0.93	48
central canal spinal cord/medulla	-1	0.94	65
fourth ventricle	-1	0.97	73
Thalamus	-1	0.97	51
corpus callosum extreme capsule	-1	0.98	16
amygdalar capsule	-1	0.98	20
Midbrain sensory related	-1	0.98	44
Cortical plate	-1	0.98	33
Cortical subplate	-1	0.99	32
anterior commissure temporal limb	-1	0.99	40
olfactory nerve layer of main olfactory bulb	-1	0.99	24
medial forebrain bundle	-1	0.99	70
solitary tract	-1	0.93	48
central canal spinal cord/medulla	-1	0.94	65

Table S4: The pattern of correlations and anticorrelations in the largest mode of variation is quantified for cortical structures at level 7 of the Allen atlas ontology. They are sorted by magnitude of log scale change and grouped by sign. Structures with the same sign are correlated with each other, and anticorrelated with those of opposite sign. Those with a negative sign are correlated with the thalamus.

structure	sign	mean scale change	mean displacement (μm)
Hippocampal region	1	1.01	40
Anterior olfactory nucleus	1	1.01	40
Main olfactory bulb	1	1.01	29
Postpiriform transition area	1	1.01	42
Piriform-amygdalar area	1	1.00	33
Retrosplenial area	-1	0.96	56
Posterior parietal association areas	-1	0.97	43
Infralimbic area	-1	0.97	40
Anterior cingulate area	-1	0.97	66
Visual areas	-1	0.97	38
Dorsal peduncular area	-1	0.97	48
Taenia tecta	-1	0.98	57
Temporal association areas	-1	0.98	24
Somatosensory areas	-1	0.98	25
Auditory areas	-1	0.98	25
Visceral area	-1	0.98	23
Ectorhinal area	-1	0.98	16
Gustatory areas	-1	0.98	20
Agranular insular area	-1	0.98	21
Orbital area	-1	0.98	27
Somatomotor areas	-1	0.98	29
Accessory olfactory bulb	-1	0.98	34
Prelimbic area	-1	0.99	33
Perirhinal area	-1	0.99	15
Hippocampal formation	-1	0.99	39
Olfactory areas	-1	0.99	29
Retrohippocampal region	-1	0.99	35
Piriform area	-1	0.99	25
Frontal pole cerebral cortex	-1	0.99	31
Cortical amygdalar area	-1	0.99	38
Nucleus of the lateral olfactory tract	-1	1.00	32
Retrosplenial area	-1	0.96	56
Posterior parietal association areas	-1	0.97	43
Infralimbic area	-1	0.97	40
Anterior cingulate area	-1	0.97	66
Visual areas	-1	0.97	38
Dorsal peduncular area	-1	0.97	48
Taenia tecta	-1	0.98	57
Temporal association areas	-1	0.98	24
Somatosensory areas	-1	0.98	25
Auditory areas	-1	0.98	25
Visceral area	-1	0.98	23
Ectorhinal area	-1	0.98	16
Gustatory areas	-1	0.98	20
Agranular insular area	-1	0.98	21
Orbital area	-1	0.98	27
Somatomotor areas	-1	0.98	29
Accessory olfactory bulb	-1	0.98	34
Prelimbic area	-1	0.99	33

2 Male only

Table S5: The relative growth corresponding to 7 weeks is quantified for all structures at level 5 of the Allen atlas ontology. They are sorted by magnitude of log scale change and grouped by sign. Scale change is relative to spatially uniform background.

structure	sign	mean scale change	mean displacement (μm)
doral tegmental decussation	1	1.08	74
medial longitudinal fascicle	1	1.07	132
cerebellar commissure	1	1.07	57
crossed tectospinal pathway	1	1.06	75
internal capsule	1	1.06	42
Fastigial nucleus	1	1.06	93
nigrostriatal tract	1	1.06	48
lateral ventricle	1	1.05	67
fiber tracts	1	1.05	46
corticospinal tract	1	1.05	43
subependymal zone	1	1.05	76
cerebal peduncle	1	1.05	50
vomeronasal nerve	1	1.05	138
middle cerebellar peduncle	1	1.05	62
optic tract	1	1.05	57
arbor vitae	1	1.05	69
motor root of the trigeminal nerve	1	1.05	65
stria terminalis	1	1.05	50
cervicothalamic tract	1	1.04	65
choroid plexus	1	1.04	82
auditory radiation	1	1.04	53
corpus callosum body	1	1.04	67
cochlear nerve	1	1.04	71
genu of corpus callosum	1	1.04	68
external medullary lamina of the thalamus	1	1.04	32
supraoptic commissures	1	1.04	37
fornix system	1	1.04	49
ventral tegmental decussation	1	1.03	78
oculomotor nerve	1	1.03	49
Medulla	1	1.03	91
cerebral aqueduct	1	1.03	128
posterior commissure	1	1.03	66
optic radiation	1	1.03	51
inferior cerebellar peduncle	1	1.03	61
facial nerve	1	1.03	120
Vermal regions	1	1.03	83
superior cerebelar peduncles	1	1.03	60
Midbrain behavioral state related	1	1.03	64
trochlear nerve	1	1.03	84
third ventricle	1	1.03	42
Midbrain motor related	1	1.03	79
Pons	1	1.03	76
Midbrain	1	1.03	72
Dentate nucleus	1	1.03	80
cingulum bundle	1	1.03	61
Cerebellum	1	1.03	70
mammillary related	1	1.03	68
Interposed nucleus	1	1.03	69
pyramid	1	1.03	46
supra-callosal cerebral white matter	1	1.03	42
optic nerve	1	1.03	30

genu of the facial nerve	1	1.03	141
vestibular nerve	1	1.02	59
corpus callosum anterior forceps	1	1.02	45
Pallidum	1	1.02	47
Hypothalamus	1	1.02	36
sensory root of the trigeminal nerve	1	1.02	57
optic chiasm	1	1.02	15
corpus callosum splenium	1	1.02	43
anterior commissure olfactory limb	1	1.02	37
rubrospinal tract	1	1.02	62
Striatum	1	1.02	49
Vestibulocerebellar nucleus	1	1.02	101
lateral recess	1	1.02	91
anterior commissure temporal limb	1	1.02	48
corpus callosum posterior forceps	1	1.02	42
superior colliculus commissure	1	1.02	75
epithalamus related	1	1.01	60
brachium of the superior colliculus	1	1.01	67
pyramidal decussation	1	1.01	54
olfactory nerve layer of main olfactory bulb	1	1.01	31
lateral olfactory tract general	1	1.01	36
medial forebrain bundle	1	1.01	62
solitary tract	-1	0.90	98
central canal spinal cord/medulla	-1	0.92	121
Thalamus	-1	0.97	60
fourth ventricle	-1	0.97	97
Hemispheric regions	-1	0.98	65
Midbrain sensory related	-1	0.98	52
Cortical plate	-1	0.98	47
corpus callosum extreme capsule	-1	0.98	20
amygdalar capsule	-1	0.98	21
Cortical subplate	-1	0.99	36
solitary tract	-1	0.90	98
central canal spinal cord/medulla	-1	0.92	121

Table S6: The relative growth corresponding to 7 weeks is quantified for cortical structures at level 7 of the Allen atlas ontology. They are sorted by magnitude of log scale change and grouped by sign. Scale change is relative to spatially uniform background.

structure	sign	mean scale change	mean displacement (μm)
Main olfactory bulb	1	1.02	44
Hippocampal region	1	1.02	62
Postpiriform transition area	1	1.02	68
Retrohippocampal region	1	1.01	58
Hippocampal formation	1	1.01	61
Piriform-amygdalar area	1	1.01	38
Infralimbic area	-1	0.95	60
Retrosplenial area	-1	0.95	64
Dorsal peduncular area	-1	0.96	71
Prelimbic area	-1	0.96	59
Posterior parietal association areas	-1	0.96	46
Anterior cingulate area	-1	0.96	96
Orbital area	-1	0.97	63
Frontal pole cerebral cortex	-1	0.97	89
Visual areas	-1	0.97	48
Taenia tecta	-1	0.98	59
Accessory olfactory bulb	-1	0.98	81
Somatosensory areas	-1	0.98	35
Auditory areas	-1	0.98	37
Temporal association areas	-1	0.98	42
Visceral area	-1	0.98	22
Somatomotor areas	-1	0.98	42
Ectorhinal area	-1	0.98	39
Gustatory areas	-1	0.99	26
Agranular insular area	-1	0.99	27
Olfactory areas	-1	0.99	38
Anterior olfactory nucleus	-1	0.99	36
Nucleus of the lateral olfactory tract	-1	0.99	42
Piriform area	-1	0.99	27
Cortical amygdalar area	-1	0.99	50
Perirhinal area	-1	0.99	38
Infralimbic area	-1	0.95	60
Retrosplenial area	-1	0.95	64
Dorsal peduncular area	-1	0.96	71
Prelimbic area	-1	0.96	59
Posterior parietal association areas	-1	0.96	46
Anterior cingulate area	-1	0.96	96
Orbital area	-1	0.97	63
Frontal pole cerebral cortex	-1	0.97	89
Visual areas	-1	0.97	48
Taenia tecta	-1	0.98	59
Accessory olfactory bulb	-1	0.98	81
Somatosensory areas	-1	0.98	35
Auditory areas	-1	0.98	37

Table S7: The pattern of correlations and anticorrelations in the largest mode of variation is quantified for all structures at level 5 of the Allen atlas ontology. They are sorted by magnitude of log scale change and grouped by sign. Structures with the same sign are correlated with each other, and anticorrelated with those of opposite sign.

structure	sign	mean scale change	mean displacement (μm)
doral tegmental decussation	1	1.08	74
medial longitudinal fascicle	1	1.07	132
cerebellar commissure	1	1.07	57
crossed tectospinal pathway	1	1.06	75
internal capsule	1	1.06	42
Fastigial nucleus	1	1.06	93
nigrostriatal tract	1	1.06	48
lateral ventricle	1	1.05	67
fiber tracts	1	1.05	46
corticospinal tract	1	1.05	43
subependymal zone	1	1.05	76
cerebal peduncle	1	1.05	50
vomeronasal nerve	1	1.05	138
middle cerebellar peduncle	1	1.05	62
optic tract	1	1.05	57
arbor vitae	1	1.05	69
motor root of the trigeminal nerve	1	1.05	65
stria terminalis	1	1.05	50
cervicothalamic tract	1	1.04	65
choroid plexus	1	1.04	82
auditory radiation	1	1.04	53
corpus callosum body	1	1.04	67
cochlear nerve	1	1.04	71
genu of corpus callosum	1	1.04	68
external medullary lamina of the thalamus	1	1.04	32
supraoptic commissures	1	1.04	37
fornix system	1	1.04	49
ventral tegmental decussation	1	1.03	78
oculomotor nerve	1	1.03	49
Medulla	1	1.03	91
cerebral aqueduct	1	1.03	128
posterior commissure	1	1.03	66
optic radiation	1	1.03	51
inferior cerebellar peduncle	1	1.03	61
facial nerve	1	1.03	120
Vermal regions	1	1.03	83
superior cerebelar peduncles	1	1.03	60
Midbrain behavioral state related	1	1.03	64
trochlear nerve	1	1.03	84
third ventricle	1	1.03	42
Midbrain motor related	1	1.03	79
Pons	1	1.03	76
Midbrain	1	1.03	72
Dentate nucleus	1	1.03	80
cingulum bundle	1	1.03	61
Cerebellum	1	1.03	70
mammillary related	1	1.03	68
Interposed nucleus	1	1.03	69
pyramid	1	1.03	46
supra-callosal cerebral white matter	1	1.03	42
optic nerve	1	1.03	30
genu of the facial nerve	1	1.03	141

vestibular nerve	1	1.02	59
corpus callosum anterior forceps	1	1.02	45
Pallidum	1	1.02	47
Hypothalamus	1	1.02	36
sensory root of the trigeminal nerve	1	1.02	57
optic chiasm	1	1.02	15
corpus callosum splenium	1	1.02	43
anterior commissure olfactory limb	1	1.02	37
rubrospinal tract	1	1.02	62
Striatum	1	1.02	49
Vestibulocerebellar nucleus	1	1.02	101
lateral recess	1	1.02	91
anterior commissure temporal limb	1	1.02	48
corpus callosum posterior forceps	1	1.02	42
superior colliculus commissure	1	1.02	75
epithalamus related	1	1.01	60
brachium of the superior colliculus	1	1.01	67
pyramidal decussation	1	1.01	54
olfactory nerve layer of main olfactory bulb	1	1.01	31
lateral olfactory tract general	1	1.01	36
medial forebrain bundle	1	1.01	62
solitary tract	-1	0.90	98
central canal spinal cord/medulla	-1	0.92	121
Thalamus	-1	0.97	60
fourth ventricle	-1	0.97	97
Hemispheric regions	-1	0.98	65
Midbrain sensory related	-1	0.98	52
Cortical plate	-1	0.98	47
corpus callosum extreme capsule	-1	0.98	20
amygdalar capsule	-1	0.98	21
Cortical subplate	-1	0.99	36
solitary tract	-1	0.90	98
central canal spinal cord/medulla	-1	0.92	121

Table S8: The pattern of correlations and anticorrelations in the largest mode of variation is quantified for cortical structures at level 7 of the Allen atlas ontology. They are sorted by magnitude of log scale change and grouped by sign. Structures with the same sign are correlated with each other, and anticorrelated with those of opposite sign. Those with a negative sign are correlated with the thalamus.

structure	sign	mean scale change	mean displacement (μm)
Main olfactory bulb	1	1.02	44
Hippocampal region	1	1.02	62
Postpiriform transition area	1	1.02	68
Retrohippocampal region	1	1.01	58
Hippocampal formation	1	1.01	61
Piriform-amygdalar area	1	1.01	38
Infralimbic area	-1	0.95	60
Retrosplenial area	-1	0.95	64
Dorsal peduncular area	-1	0.96	71
Prelimbic area	-1	0.96	59
Posterior parietal association areas	-1	0.96	46
Anterior cingulate area	-1	0.96	96
Orbital area	-1	0.97	63
Frontal pole cerebral cortex	-1	0.97	89
Visual areas	-1	0.97	48
Taenia tecta	-1	0.98	59
Accessory olfactory bulb	-1	0.98	81
Somatosensory areas	-1	0.98	35
Auditory areas	-1	0.98	37
Temporal association areas	-1	0.98	42
Visceral area	-1	0.98	22
Somatomotor areas	-1	0.98	42
Ectorhinal area	-1	0.98	39
Gustatory areas	-1	0.99	26
Agranular insular area	-1	0.99	27
Olfactory areas	-1	0.99	38
Anterior olfactory nucleus	-1	0.99	36
Nucleus of the lateral olfactory tract	-1	0.99	42
Piriform area	-1	0.99	27
Cortical amygdalar area	-1	0.99	50
Perirhinal area	-1	0.99	38
Infralimbic area	-1	0.95	60
Retrosplenial area	-1	0.95	64
Dorsal peduncular area	-1	0.96	71
Prelimbic area	-1	0.96	59
Posterior parietal association areas	-1	0.96	46
Anterior cingulate area	-1	0.96	96
Orbital area	-1	0.97	63
Frontal pole cerebral cortex	-1	0.97	89
Visual areas	-1	0.97	48
Taenia tecta	-1	0.98	59
Accessory olfactory bulb	-1	0.98	81
Somatosensory areas	-1	0.98	35
Auditory areas	-1	0.98	37

3 Female only

Table S9: The relative growth corresponding to 7 weeks is quantified for all structures at level 5 of the Allen atlas ontology. They are sorted by magnitude of log scale change and grouped by sign. Scale change is relative to spatially uniform background.

structure	sign	mean scale change	mean displacement (μm)
cerebellar commissure	1	1.05	42
cerebal peduncle	1	1.04	40
doral tegmental decussation	1	1.04	48
Fastigial nucleus	1	1.04	60
internal capsule	1	1.04	42
medial longitudinal fascicle	1	1.04	62
corticospinal tract	1	1.04	21
crossed tectospinal pathway	1	1.03	30
nigrostriatal tract	1	1.03	49
arbor vitae	1	1.03	40
oculomotor nerve	1	1.03	40
motor root of the trigeminal nerve	1	1.03	30
middle cerebellar peduncle	1	1.03	31
fiber tracts	1	1.03	42
cervicothalamic tract	1	1.03	45
facial nerve	1	1.03	53
auditory radiation	1	1.03	40
lateral ventricle	1	1.03	35
stria terminalis	1	1.03	44
subependymal zone	1	1.03	38
choroid plexus	1	1.03	43
Vermal regions	1	1.02	47
corpus callosum body	1	1.02	37
external medullary lamina of the thalamus	1	1.02	38
Medulla	1	1.02	46
Hemispheric regions	1	1.02	46
optic tract	1	1.02	54
cochlear nerve	1	1.02	36
trochlear nerve	1	1.02	32
inferior cerebellar peduncle	1	1.02	28
Interposed nucleus	1	1.02	29
superior cerebelar peduncles	1	1.02	25
ventral tegmental decussation	1	1.02	68
Cerebellum	1	1.02	36
Pons	1	1.02	30
Midbrain motor related	1	1.02	48
fornix system	1	1.02	28
Midbrain	1	1.02	51
Midbrain behavioral state related	1	1.02	36
supra-callosal cerebral white matter	1	1.02	23
optic nerve	1	1.02	32
optic radiation	1	1.02	28
Vestibulocerebellar nucleus	1	1.02	61
Dentate nucleus	1	1.02	43
vestibular nerve	1	1.02	19
lateral olfactory tract general	1	1.02	44
corpus callosum splenium	1	1.02	20
lateral recess	1	1.02	37
rubrospinal tract	1	1.02	37
genu of the facial nerve	1	1.02	64
third ventricle	1	1.02	35

genu of corpus callosum	1	1.01	33
posterior commissure	1	1.01	34
cerebral aqueduct	1	1.01	76
Hypothalamus	1	1.01	47
pyramid	1	1.01	26
Pallidum	1	1.01	42
cingulum bundle	1	1.01	34
sensory root of the trigeminal nerve	1	1.01	28
anterior commissure olfactory limb	1	1.01	56
corpus callosum posterior forceps	1	1.01	20
brachium of the superior colliculus	1	1.01	31
supraoptic commissures	1	1.01	38
vomeronasal nerve	1	1.01	65
optic chiasm	1	1.01	23
superior colliculus commissure	1	1.01	41
solitary tract	-1	0.94	38
central canal spinal cord/medulla	-1	0.96	40
corpus callosum extreme capsule	-1	0.97	19
fourth ventricle	-1	0.97	66
amygdalar capsule	-1	0.97	26
Thalamus	-1	0.98	50
mammillary related	-1	0.98	57
Cortical subplate	-1	0.98	37
Cortical plate	-1	0.98	36
Midbrain sensory related	-1	0.99	44
corpus callosum anterior forceps	-1	0.99	24
Striatum	-1	0.99	36
anterior commissure temporal limb	-1	0.99	39
medial forebrain bundle	-1	0.99	77
olfactory nerve layer of main olfactory bulb	-1	0.99	39
epithalamus related	-1	0.99	47
pyramidal decussation	-1	1.00	20
solitary tract	-1	0.94	38

Table S10: The relative growth corresponding to 7 weeks is quantified for cortical structures at level 7 of the Allen atlas ontology. They are sorted by magnitude of log scale change and grouped by sign. Scale change is relative to spatially uniform background.

structure	sign	mean scale change	mean displacement (μm)
Anterior olfactory nucleus	1	1.02	57
Frontal pole cerebral cortex	1	1.01	58
Hippocampal region	1	1.01	35
Retrosplenial area	-1	0.96	52
Posterior parietal association areas	-1	0.97	40
Visual areas	-1	0.97	38
Anterior cingulate area	-1	0.98	56
Temporal association areas	-1	0.98	28
Ectorhinal area	-1	0.98	23
Visceral area	-1	0.98	30
Agranular insular area	-1	0.98	34
Infralimbic area	-1	0.98	27
Gustatory areas	-1	0.98	24
Dorsal peduncular area	-1	0.98	34
Somatosensory areas	-1	0.98	24
Taenia tecta	-1	0.98	58
Auditory areas	-1	0.98	27
Perirhinal area	-1	0.98	21
Somatomotor areas	-1	0.98	30
Main olfactory bulb	-1	0.99	51
Accessory olfactory bulb	-1	0.99	56
Orbital area	-1	0.99	38
Retrohippocampal region	-1	0.99	32
Piriform area	-1	0.99	34
Hippocampal formation	-1	0.99	33
Olfactory areas	-1	0.99	42
Prelimbic area	-1	0.99	38
Cortical amygdalar area	-1	0.99	38
Postpiriform transition area	-1	0.99	22
Piriform-amygdalar area	-1	0.99	37
Nucleus of the lateral olfactory tract	-1	0.99	43
Retrosplenial area	-1	0.96	52
Posterior parietal association areas	-1	0.97	40
Visual areas	-1	0.97	38
Anterior cingulate area	-1	0.98	56
Temporal association areas	-1	0.98	28
Ectorhinal area	-1	0.98	23
Visceral area	-1	0.98	30
Agranular insular area	-1	0.98	34
Infralimbic area	-1	0.98	27
Gustatory areas	-1	0.98	24
Dorsal peduncular area	-1	0.98	34
Somatosensory areas	-1	0.98	24
Taenia tecta	-1	0.98	58
Auditory areas	-1	0.98	27
Perirhinal area	-1	0.98	21

Table S11: The pattern of correlations and anticorrelations in the largest mode of variation is quantified for all structures at level 5 of the Allen atlas ontology. They are sorted by magnitude of log scale change and grouped by sign. Structures with the same sign are correlated with each other, and anticorrelated with those of opposite sign.

structure	sign	mean scale change	mean displacement (μm)
cerebellar commissure	1	1.05	42
cerebal peduncle	1	1.04	40
doral tegmental decussation	1	1.04	48
Fastigial nucleus	1	1.04	60
internal capsule	1	1.04	42
medial longitudinal fascicle	1	1.04	62
corticospinal tract	1	1.04	21
crossed tectospinal pathway	1	1.03	30
nigrostriatal tract	1	1.03	49
arbor vitae	1	1.03	40
oculomotor nerve	1	1.03	40
motor root of the trigeminal nerve	1	1.03	30
middle cerebellar peduncle	1	1.03	31
fiber tracts	1	1.03	42
cervicothalamic tract	1	1.03	45
facial nerve	1	1.03	53
auditory radiation	1	1.03	40
lateral ventricle	1	1.03	35
stria terminalis	1	1.03	44
subependymal zone	1	1.03	38
choroid plexus	1	1.03	43
Vermal regions	1	1.02	47
corpus callosum body	1	1.02	37
external medullary lamina of the thalamus	1	1.02	38
Medulla	1	1.02	46
Hemispheric regions	1	1.02	46
optic tract	1	1.02	54
cochlear nerve	1	1.02	36
trochlear nerve	1	1.02	32
inferior cerebellar peduncle	1	1.02	28
Interposed nucleus	1	1.02	29
superior cerebelar peduncles	1	1.02	25
ventral tegmental decussation	1	1.02	68
Cerebellum	1	1.02	36
Pons	1	1.02	30
Midbrain motor related	1	1.02	48
fornix system	1	1.02	28
Midbrain	1	1.02	51
Midbrain behavioral state related	1	1.02	36
supra-callosal cerebral white matter	1	1.02	23
optic nerve	1	1.02	32
optic radiation	1	1.02	28
Vestibulocerebellar nucleus	1	1.02	61
Dentate nucleus	1	1.02	43
vestibular nerve	1	1.02	19
lateral olfactory tract general	1	1.02	44
corpus callosum splenium	1	1.02	20
lateral recess	1	1.02	37
rubrospinal tract	1	1.02	37
genu of the facial nerve	1	1.02	64
third ventricle	1	1.02	35
genu of corpus callosum	1	1.01	33

posterior commissure	1	1.01	34
cerebral aqueduct	1	1.01	76
Hypothalamus	1	1.01	47
pyramid	1	1.01	26
Pallidum	1	1.01	42
cingulum bundle	1	1.01	34
sensory root of the trigeminal nerve	1	1.01	28
anterior commissure olfactory limb	1	1.01	56
corpus callosum posterior forceps	1	1.01	20
brachium of the superior colliculus	1	1.01	31
supraoptic commissures	1	1.01	38
vomeronasal nerve	1	1.01	65
optic chiasm	1	1.01	23
superior colliculus commissure	1	1.01	41
solitary tract	-1	0.94	38
central canal spinal cord/medulla	-1	0.96	40
corpus callosum extreme capsule	-1	0.97	19
fourth ventricle	-1	0.97	66
amygdalar capsule	-1	0.97	26
Thalamus	-1	0.98	50
mammillary related	-1	0.98	57
Cortical subplate	-1	0.98	37
Cortical plate	-1	0.98	36
Midbrain sensory related	-1	0.99	44
corpus callosum anterior forceps	-1	0.99	24
Striatum	-1	0.99	36
anterior commissure temporal limb	-1	0.99	39
medial forebrain bundle	-1	0.99	77
olfactory nerve layer of main olfactory bulb	-1	0.99	39
epithalamus related	-1	0.99	47
pyramidal decussation	-1	1.00	20
solitary tract	-1	0.94	38

Table S12: The pattern of correlations and anticorrelations in the largest mode of variation is quantified for cortical structures at level 7 of the Allen atlas ontology. They are sorted by magnitude of log scale change and grouped by sign. Structures with the same sign are correlated with each other, and anticorrelated with those of opposite sign. Those with a negative sign are correlated with the thalamus.

structure	sign	mean scale change	mean displacement (μm)
Anterior olfactory nucleus	1	1.02	57
Frontal pole cerebral cortex	1	1.01	58
Hippocampal region	1	1.01	35
Retrosplenial area	-1	0.96	52
Posterior parietal association areas	-1	0.97	40
Visual areas	-1	0.97	38
Anterior cingulate area	-1	0.98	56
Temporal association areas	-1	0.98	28
Ectorhinal area	-1	0.98	23
Visceral area	-1	0.98	30
Agranular insular area	-1	0.98	34
Infralimbic area	-1	0.98	27
Gustatory areas	-1	0.98	24
Dorsal peduncular area	-1	0.98	34
Somatosensory areas	-1	0.98	24
Taenia tecta	-1	0.98	58
Auditory areas	-1	0.98	27
Perirhinal area	-1	0.98	21
Somatomotor areas	-1	0.98	30
Main olfactory bulb	-1	0.99	51
Accessory olfactory bulb	-1	0.99	56
Orbital area	-1	0.99	38
Retrohippocampal region	-1	0.99	32
Piriform area	-1	0.99	34
Hippocampal formation	-1	0.99	33
Olfactory areas	-1	0.99	42
Prelimbic area	-1	0.99	38
Cortical amygdalar area	-1	0.99	38
Postpiriform transition area	-1	0.99	22
Piriform-amygdalar area	-1	0.99	37
Nucleus of the lateral olfactory tract	-1	0.99	43
Retrosplenial area	-1	0.96	52
Posterior parietal association areas	-1	0.97	40
Visual areas	-1	0.97	38
Anterior cingulate area	-1	0.98	56
Temporal association areas	-1	0.98	28
Ectorhinal area	-1	0.98	23
Visceral area	-1	0.98	30
Agranular insular area	-1	0.98	34
Infralimbic area	-1	0.98	27
Gustatory areas	-1	0.98	24
Dorsal peduncular area	-1	0.98	34
Somatosensory areas	-1	0.98	24
Taenia tecta	-1	0.98	58
Auditory areas	-1	0.98	27
Perirhinal area	-1	0.98	21

Supplementary Note 7: Code documentation

GDM

Release 0.0.1

Daniel Tward, Bryson Gray

Feb 12, 2024

CONTENTS:

1 Introduction 3

2 Installation 9

3 Coordinate Systems 11

4 File Formats 19

5 Input specification via Transformation Graph Interface 23

6 Output Specification 27

7 Examples 29

8 Function reference 49

9 Work in progress 51

10 Installing 53

11 Examples 55

12 Important Functions 57

13 Module and function documentation 59

14 Web interface 61

Generative diffeomorphic mapping (GDM) is a deformable image registration algorithm designed for aligning multi-modal neuroimaging datasets to one another for subsequent analysis. Our package has several important novel features including estimation of any differences in contrast or color between datasets, identification of missing tissues or artifactions, diverse geometries such as mapping 3D volumes to a sequence of 2D datasets, and complex multimodality registration setups described by transformation graphs.

This documentation can automatically be rendered as a pdf using the sphinx package but is best viewed in html (https://twardlab.github.io/emlddmm/build/html/index.html). The pdf may have missing or broken links and images.

CHAPTER ONE

INTRODUCTION

The purpose of our pipeline is to coregister neuroimaging datasets of different modalities and with different coordinate systems. We support 3D to 3D mapping, 3D to 2D mapping (e.g. mapping to serial sections), and 2D to 2D mapping (e.g. rigidly aligning slices with different stains).

We perform registration using diffeomorphisms (with time varying velocity field parameterization) and affine transforms. These transformations can be composed to map data between coordinate spaces and between single specimens and common coordinate systems.

Examples of typical workflows are below. In the diagrams below, each arrow represents the computation of a transformation. By following arrows in the forward or reverse direction, all data can be reconstructed in any of the available spaces. A minor caveat is that only low resolution 2D summary data can be reconstructed in a 3D space.

1.1 Example workflow: STP mapping

A common setting is when we do not have serial section data. For example we may map the Allen atlas to a single 3D STP image. We will need to superimpose atlas labels on the STPT image, and transform the STPT image to match the shape of the atlas.

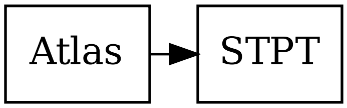

Fig. 1: An example task of 3D to 3D mapping between an atlas and a Serial Two Photon Tomography dataset.

1.2 Example workflow: Alternating sections to atlases

A typical example is to image a mouse brain using serial sections. Alternate sections are stained for Nissl, or for a specific fluorescent tracer. The pipeline will rigidly register fluorescent slices to neighboring Nissl slices, and will deformably register the Allen CCF Nissl atlas onto the 3D stack of Nissl slices. This allows us to map the anatomical labels from the atlas onto our slices. On each slice, we can quantify cell counts or fluorescence in atlas regions. In 3D we can quantify tracer or cell density.

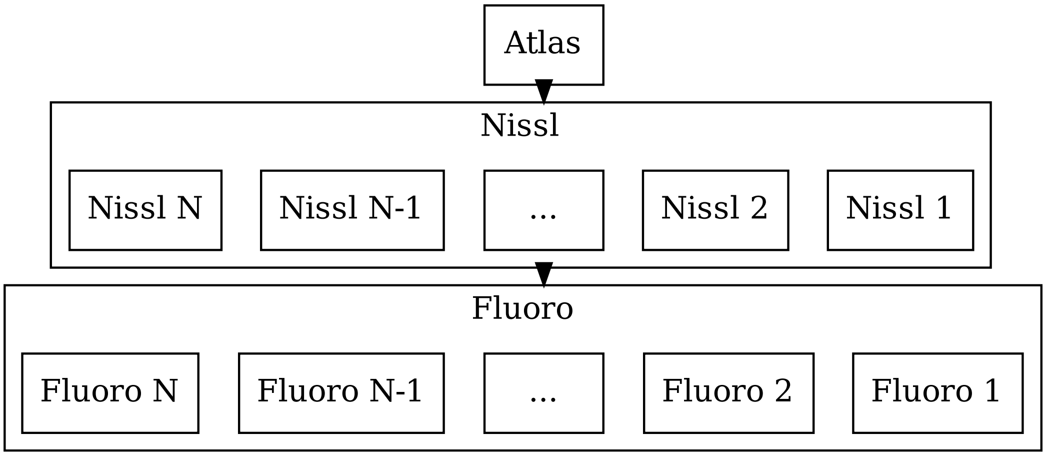

Fig. 2: We map our 3D atlas onto a series of 2D nissl images. We also map our 2D Nissl images to their nearest fluorescent image

Note that any time our pipeline registers a 3D volume to a set of 2D slices, a new space is automatically created called a "registered" space. In this space, all the Nissl sections will be rigidly aligned into a 3D reconstruction.

1.3 Example workflow: Ex vivo MRI

Another example is when MRI is available for a specimen. We typically have an in vivo MRI, ex vivo MRI, and serial section microscopy. The registration tasks are: i) ex vivo to in vivo, ii) ex vivo to serial sections, iii) ex vivo to atlas. We may wish to reconstruct our data in any of the three spaces (in vivo, ex vivo, or atlas). Here the ex vivo MRI plays the role of a common space that is mapped to everything.

Again, a reconstructed space will be automatically created.

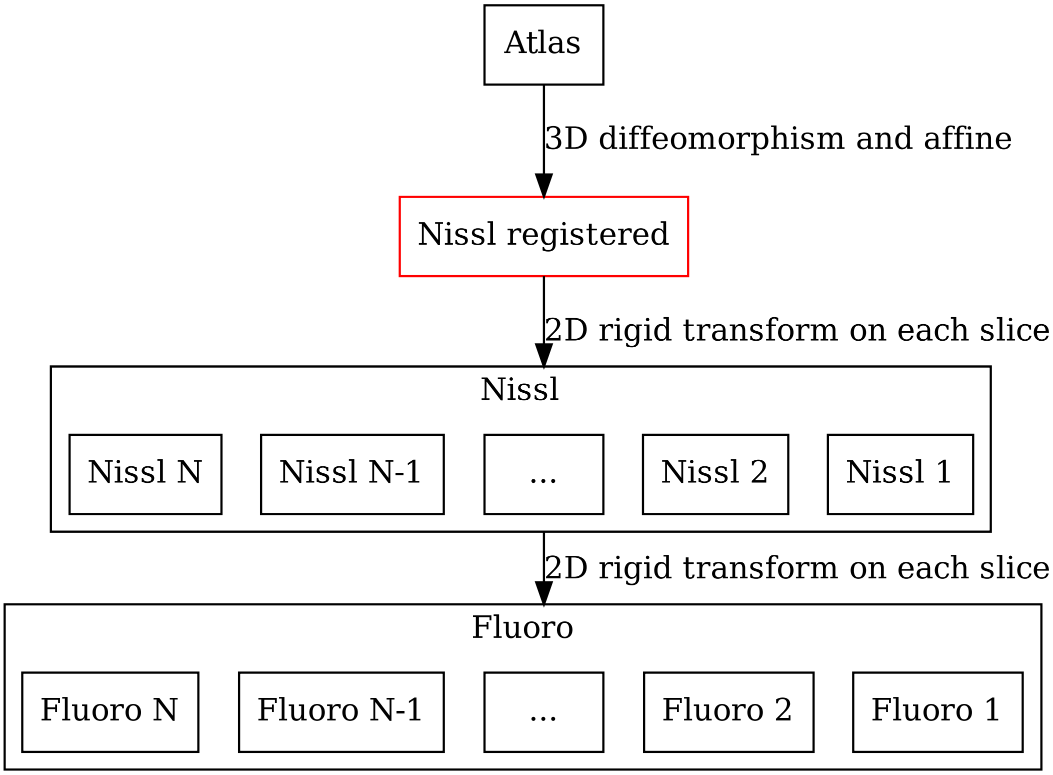

Fig. 3: For any 3D to 2D map, a registered space is automatically created (shown in red). No input data is associated with this space, but images can be reconstructed into this space.

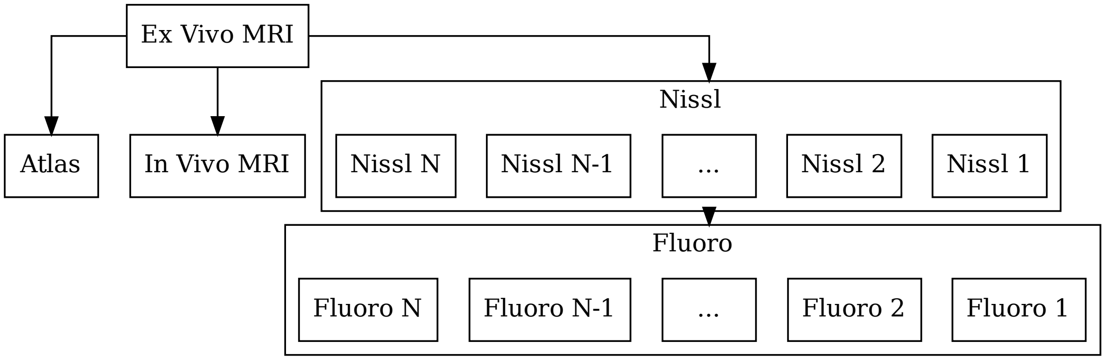

Fig. 4: We may also include in vivo and ex vivo mri.

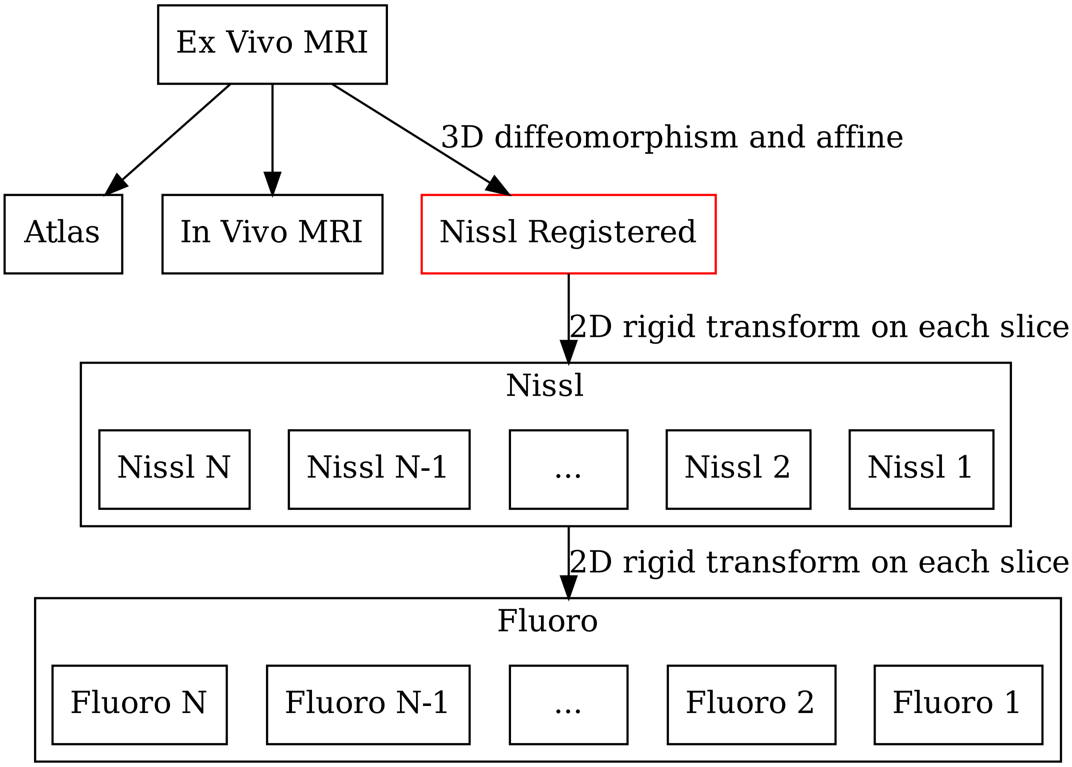

Fig. 5: For any 3D to 2D map, a registered space is automatically created (shown in red). No input data is associated with this space, but images can be reconstructed into this space.

1.4 Example workflow: Arbitrary layout

In general, a registration task can be formulated by a directed acyclic graph. Each node in the graph is a “space”, which may have more than one image associated with it. Each arrow in the graph is a registration task.

We have built infrastructure to perform necessary maps, and compose transforms to reconstruct any dataset in any space.

CHAPTER

TWO

INSTALLATION

Clone the repository:

```
git clone github.com/twardlab/emlddmm
```

Change to the directory emlddmm has been cloned to and install the requirements:

```
pip install -r requirements.txt
```

When running interactively in python, add the appropriate path:

```
import sys
sys.path.append('PATH_TO_EMLDDMM_LIBRARY')
```

When running from command line:

```
python PATH_TO_EMLDDMM_LIBRARY/transformation_graph_v01.py --in INPUT_FILENAME
```

For details on the command line interface, see *input specification*.

CHAPTER

THREE

COORDINATE SYSTEMS

Our pipeline computes transformations between pairs of spaces. Each space can include one or more images that are sampled on the same voxel grid. Spaces are defined by an origin and an orientation, and often a voxel size. Depending on conventions, these may be specified relative to anatomy in an image (common for atlases) or relative to the sampling grid the image was obtained on. The latter case is common because generally we don't know where the anatomy is until after we have solved a registeration problem.

3.1 Atlas spaces

Coordinate systems associated with several different atlases are described here.

3.1.1 Mouse atlas

We adopt the Allen Institute's Common Coordinate Framework (CCF) atlas, and use its structure annotations, its 3D Nissl image, and its 3D STPT average template image.

Note that the STPT image and annotations are left right symmetric in this data. Nissl images are not left right symmetric.

The CCF NRRD files do not specify a coordinate system correctly. We build our own right handed coordinate system which is described below.

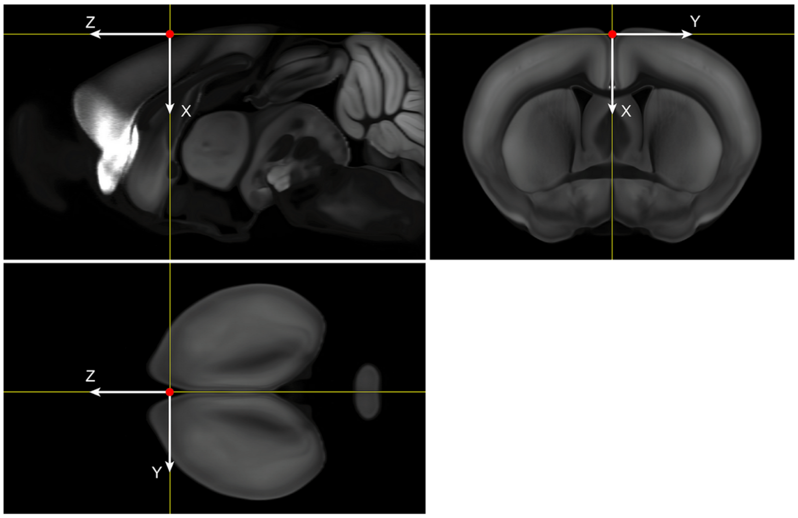

Coordinate directions

We chose "x" to point from the brain's superior to inferior, "y" to point from the brain's left to right (note this is often displayed by pointing from the right side of the screen to the left, i.e. radiological convention), and "z" to point from posterior to anterior (caudal to rostral).

Coordinate origin

We chose "x" to point from the brain's superior to inferior, "y" to point from the brain's left to right (note this is often displayed by pointing from the right side of the screen to the left, i.e. radiological convention), and "z" to point from posterior to anterior (caudal to rostral).

We choose the coordinate origin to be our best estimate of the bregma location, based on previous mappings to MR data with skull. Location on Average Template (volume size 8 x 11.4 x 13.2 mm): * x=930 µm from dorsal end of the volume * y=5700 µm from right end of the volume * z=8000 µm from posterior end of the volume

Pixel size

This dataset is available in 10, 25, 50, and 100 micron isotropic voxel size. We typically perform registration at 50 microns.

Other information

Warning: Different versions of annotations are available. Unless otherwise specified, we use CCF version 3. This can be downloaded here: http://download.alleninstitute.org/informatics-archive/current-release/mouse_ccf/annotation/ccf_2017/ Note, as of March 2023, there is a new version of annotations called 2022. But we are not using that version.

Note: On CSH server: /nfs/data/main/M32/RegistrationData/ATLAS/annotation_50_bregma_LR.vtk /nfs/data/main/M32/RegistrationData/ATLAS/ara_nissl_50_bregma.vtk /nfs/data/main/M32/RegistrationData/ATLAS/average_template

3.1.2 Marmoset atlas

For marmoset we typically use the RIKEN atlas described in Woodward 2018 (https://www.nature.com/articles/sdata20189). An image of our coordinate system convention is shown below.

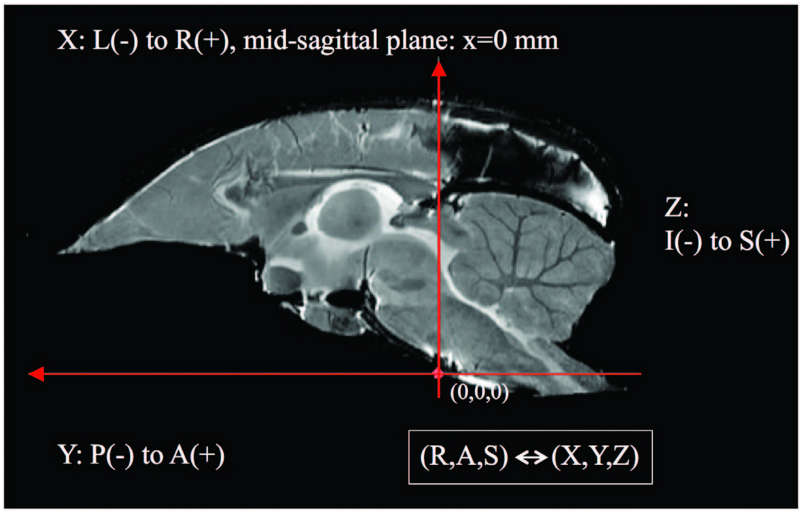

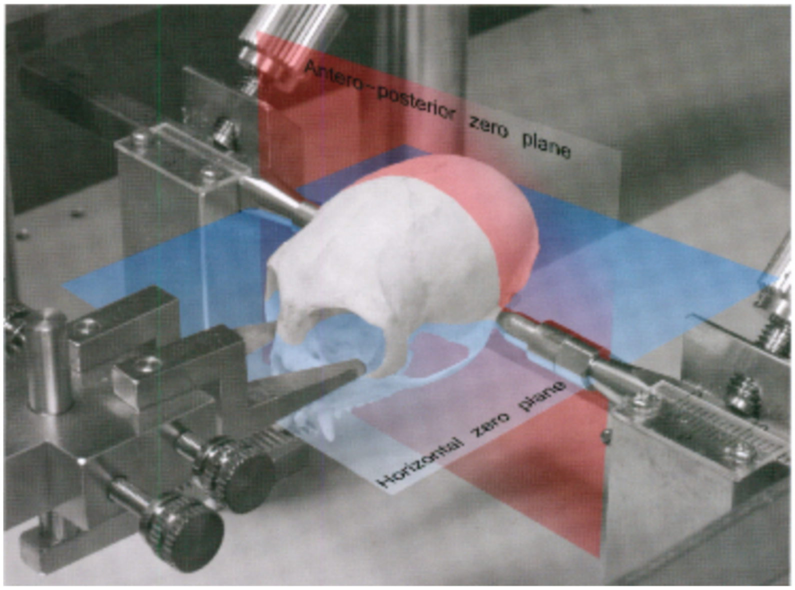

Coordinate directions

We use an RAS (right, anterior, superior) coordinate system where the "x" direction points from left to right, the "y" direction points from posterior to anterior, and the "z" direction points from inferior to superior.

Coordinate origin

In the common marmoset, the horizontal zero plane is defined as the plane passing through the lower margin of the orbit and the center of the external auditory meatus (see figure) (note you cannot see bregma on marmoset, it is fused too tightly to see). In an imaging apparatus, the skull is fixed through the ears, so this is a good choice. The anteroposterior zero plane is defined as the plane perpendicular to the horizontal zero plane which passes the centers of the external auditory meati. The left-right zero plane is the midsagittal plane (Saavedra and Mazzuchelli, 1969; Stephan et al., 1980).

Other information

Note: On our CSH dropbox system the atlas data is located here: https://www.dropbox.com/sh/70hg40e8b3ro9vx/AACk05Hm-BFGbMD5NIQolqtxa?dl=0 with images:

Nissl reference: bma-1-nissl.nii.gz

MRI reference: bma-1-mri.nii.gz

Atlas: bma-1-region_seg.nii.gz

Note: We have built a population average image for males and females, which is located on CSH at /nfs/data/main/M38/marmoset_ccf

3.1.3 Human atlas

We use the Montreal Neurological Institute - International Consortium for Brain Mapping (MNI-ICBM) coordinate system, which is described here: https://www.mcgill.ca/bic/software/tools-data-analysis/anatomical-mri/atlases/icbm152-non-linear . An example is shown below.

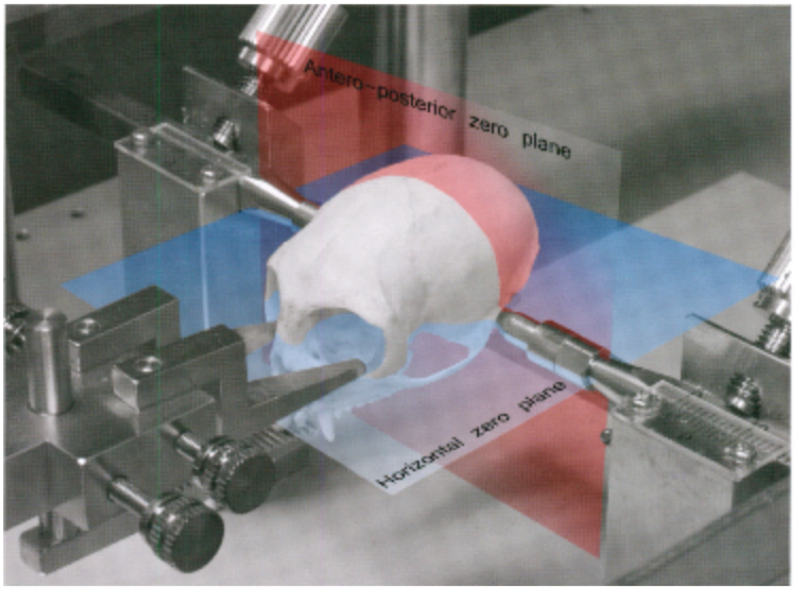

Coordinate directions

The atlas is based on the Talarach Tournoux coordinate system which is RAS. "x" points from left to right, "y" points from posterior to anterior (posterior commissure to anterior commissure), and "z" points from inferior to superior.

Coordinate origin

The coordinate origin is 4 millimeters above the center of the anterior commissure.

Details: these points are intersection of planes. AC to PC defines a line. Left right defines a line. These two lines define a plane.

Midsagittal plane defines another plane.

The intersection defines a line, and the left right center on this line gives the origin.

Voxel size

Standard voxel size is 1mm isotropic, but other possibilities are available.

Other information

Currently can be downloaded form the web at the above link https://www.mcgill.ca/bic/software/tools-data-analysis/anatomical-mri/atlases/icbm152-non-linear .

Note: Another possibility is MNI-Colin27 and not ICBM152 nonlinear. https://www.mcgill.ca/bic/software/tools-data-analysis/anatomical-mri/atlases/colin-27-2008

Note that the difference is the 4mm offset.

3.2 Input space

The input space describes the coordinate system of 2D serial section images to be registered. This is the internal structure of the pipeline, but preprocessors may be used to convert data with other structures. For example, point sets may be described by the integer index of a pixel in an image, rather than a location with units of length. A converter is necessary in this case (for example `emlddmm.convert_points_from_json()`)

3.2.1 Coordinate directions

The "x" coordinate points from left to right (i.e. from the left side to the right side of a 2D image displayed on a screen). The "y" coordinate points from up to down. The "z" coordinate points from the first acquired slice to the last acquired slice.

Note: This convention does not reference any anatomy, only the camera. This is chosen because sections could have any orientation (coronal, sagittal, etc.).

3.2.2 Coordinate origin

The xy origin will always be in the center of the image. i.e. On a given slice if you find the average "x" coordinate, or the average "y" coordinate, it will be 0. The z coordinate origin is also chosen such that slices are centered for this dataset: the average of the z coordinate for the first and last slices is zero.

Note: Motivation for this choice is that we can pad images symmetrically without changing the origin, and rotation about this origin is more numerically stable than rotation around one corner.

3.2.3 Voxel size

Voxel size is an input parameter to the pipeline (e.g. stored in json sidecar files for 2D images, or in vtk headers for 3D images). Our convention is to use units of microns.

Note: In a typical workflow, an image is created with resolution 0.46umx0.46micron, with a slice thickness of 10 micron. For registration purposes these are typically downsampled by a factor of 32 in the x and y directions, making the resolution 14.72um before they are input to the pipeline.

3.3 Registered space

In our workflow, any time a 3D to sequence of 2D slices map is calculated, a new registered space is created (see for example *here*). A sequence of rigid transformations are applied to each 2D slice to match the general shape of a deformed reference 3D image. This effectively defines a 3D coordinate space.

3.3.1 Coordinate directions

Same as *input space directions*.

3.3.2 Coordinate origin

Same as *input space origin*.

3.3.3 Voxel size

Same as *input space voxel size*.

Note: Once transformations have been computed, high resolution data is transformed into registered space for display on the web or other viewers. This high resolution data uses its native voxel size (typically 0.46 microns).

3.3.4 The non-uniqueness of registered space

Our mapping algorithm enforces alignment between data in the common space, and data in the input space. This sequence of transforms can be factored to define a space in the "middle". This is a factorization of transformations problem. Just like matrix factorizations are not unique without constraints, the data does not uniquely define a registered space. Rather, we use several heuristics to define a space that is a "minimally distorted" version of the common space. The general idea is that any component of the transform that can be represented as a sequence of 2D transforms, should be represented that way, rather than as part of a 3D transform. In particular

- We want no translation in the xy direction of the 3D affine transformation. (this is enforced by our pipeline)
- We want no shear perpendicular to the z axis in the 3D transformation. (this can be enforced by chosing to limit the affine transformation to rigid, or rigid plus scale)
- After applying our affine transform we want the up vector to still point up, when projected into the slice plane. (implemented with the `up_vector` parameter in `emlddmm.emlddmm()`)

3.3.5 Other information

While registered space uses the same coordinate system conventions as input space, the set of voxel locations images should be sampled on is likely to be different. One example occurs when our atlas has its origin at the bregma point on the skull, but our input data has its origin in the center of the brain tissue. Due to our factorization conventions described *above*, tissue in registered space will no longer be centered at the origin.

In a typical situation, our input histology is sectioned in either the coronal, sagittal, or transverse plane, and we map it to a well characterized atlas. In these situations, the origin in xy for the registered space can be interpretted with respect to the atlas origin, and will correspond to the origin for two of the 3 axes in the atlas. We enumerate sevaral cases below and provide some information explicitly.

Mouse with coronal sections

Using our *Mouse atlas*, and a coronally sectioned dataset in *Input space*, input space x corresponds to the right left axis, and input space y corresponds to the dorsal ventral axis.

Therefore, the x=0 point in registered space corresponds to the anatomy at the y=0 point in the atlas, and the y=0 point in registered space corresponds to the anatomy at the x=0 point in the atlas.

When reconstructing imaging data in this space we chose a set of sample points for voxels that will cover the anatomy. Therefore, we sample x starting at -5695.0 um, ending at 5695.06, and using 24762 samples equally spaced by 0.46 microns. Similarly, we sample y starting at -870.0 um, ending at 7120.2 um, and using 17371 samples equally spaced by 0.46 microns.

This convention allows us to convert between spatial locations and pixel indices (row=i,col=j, starting at 0), using the follow formulas:

$$i = \text{round}[(y - (-870.0))/0.46]$$
$$j = \text{round}[(x - (-5695.0))/0.46]$$
$$y = 0.46i + (-870.0)$$
$$x = 0.46j + (-5695.0)$$

Mouse with sagittal sections

Using our *Mouse atlas*, and a sagittaly sectioned dataset in *Input space*, input space x corresponds to the anterior posterior axis (note the nose will be on the left), and input space y corresponds to the dorsal ventral axis.

Therefore, the x=0 point in registered space corresponds to the anatomy at the z=0 point in the atlas, and the y=0 point in registered space corresponds to the anatomy at the x=0 point in the atlas.

When reconstructing imaging data in this space we chose a set of sample points for voxels that will cover the anatomy. Therefore, we sample x starting at -7970.0 um, ending at 5220.04, and using 28675 samples equally spaced by 0.46 microns. Similarly, we sample y starting at -870.0 um, ending at 7120.2 um, and using 17371 samples equally spaced by 0.46 microns.

This convention allows us to convert between spatial locations and pixel indices (row=i,col=j, starting at 0), using the follow formulas:

$$i = \text{round}[(y - (-870.0))/0.46]$$
$$j = \text{round}[(x - (-7970.0))/0.46]$$
$$y = 0.46i + (-870.0)$$
$$x = 0.46j + (-7970.0)$$

Mouse with transverse sections

Using our *Mouse atlas*, and a transverse sectioned dataset in *Input space*, input space x corresponds to the anterior posterior axis (CHECK!), and input space y corresponds to the right left axis.

Therefore, the x=0 point in registered space corresponds to the anatomy at the z=0 point in the atlas, and the y=0 point in registered space corresponds to the anatomy at the y=0 point in the atlas.

When reconstructing imaging data in this space we chose a set of sample points for voxels that will cover the anatomy. Therefore, we sample x starting at -7970.0 um, ending at 5220.04, and using 28675 samples equally spaced by 0.46 microns. Similarly, we sample y starting at -5695.0 um, ending at 5695.09 um, and using 24762 samples equally spaced by 0.46 microns.

This convention allows us to convert between spatial locations and pixel indices (row=i,col=j, starting at 0), using the follow formulas:

$$i = \text{round}[(y - (-5695.0))/0.46]$$
$$j = \text{round}[(x - (-7970.0))/0.46]$$
$$y = 0.46i + (-5695.0)$$
$$x = 0.46j + (-7970.0)$$

CHAPTER

FOUR

FILE FORMATS

We propose to use VTK formatted data whenever possible. Currently we use simple legacy .vtk files (https://docs.vtk.org/en/latest/design_documents/VTKFileFormats.html). This supports vector and raster graphics, works well with visualization software including web viewers, and is largely human readable. It does not support compression, and so other formats are also used.

4.1 JSON geometry files

Every imaging file that does not store geometry information (e.g. 2D slices stored as pngs/tifs/etc) will have a corresponding short JSON sidecar file located in the same directory, with the same name filename, and the extension .json appended. The information stored here should be as close as possible to an NRRD header (http://teem.sourceforge.net/nrrd/format.html). Note that 2D images are described as though they are 3D, with pixel size in the z dimension referring to section thickness.

Such sidecar files are inspired by the BIDS standard, and contain information typically stored in an NRRD header. Each sidecar file must contain the following fields:

- "DataFile": the image file name
- "SpaceDirections": a list of vectors for each image dimension specifying the unit conversion from pixel indices (row,column) to input space coordinates. Note that the z-coordinate conversion indicates the slice thickness plus the spacing between slices. This is a list of vectors in x,y,z order, where the xyz coordinate system is defined in the "input space" section.
- "SpaceOrigin": The world coordinate of the image origin, in x,y,z order.

Other metadata can be stored in the json file, but only the above 3 are generally used for the data loader functions.

Note that each 2D image is modeled as a 3D image with a single slice (i.e. a size of 1 in).

An example is shown below:

```
{
  "DataFile": "MD787_small_nissl/MD787-N27-2019.03.28-22.55.54_MD787_2_0080.png",
  "Type": "Float32",
  "Dimension": 3,
  "Endian": "big",
  "Sizes": [3, 392, 480, 1],
  "Space": "inferior-right-posterior",
  "SpaceDimension": 3,
  "SpaceUnits": ["um", "um","um" ],
  "SpaceDirections": [
    "none",
```

(continues on next page)

(continued from previous page)

```
    [44.160000000000004, 0.0, 0.0],
        [0.0, 44.160000000000004, 0.0],
        [0.0, 0.0, 200      ]
  ],
  "SliceThickness" : 10.0
  "SpaceOrigin": [-8633.28, -10576.32, -120100.0]
}
```

Note when data is read, the space directions are reversed to line up with common conventions for image array axes. The last axis of an image array corresponds to columns (x), the second last corresponds to rows (y), and the third last corresponds to slices (z).

4.2 Dataset lists

Since sections may be missing or require other comments, we include a tsv file in the same directory describing every slice in the dataset. The required fields are sample_id and status, the latter should contain present or absent. An example is shown below:

```
sample_id    participant_id          species          status
MD787-N7-2019.03.28-22.05.43_MD787_2_0020.png       MD787   Mus Musculus    present
MD787-N14-2019.03.28-22.20.46_MD787_1_0040.png      MD787   Mus Musculus    present
MD787-N20-2019.03.28-22.36.39_MD787_3_0060.png      MD787   Mus Musculus    present
MD787-N27-2019.03.28-22.55.54_MD787_2_0080.png      MD787   Mus Musculus    present
MD787-N34-2019.03.28-23.15.58_MD787_1_0100.png      MD787   Mus Musculus    present
MD787-N40-2019.03.28-23.33.43_MD787_3_0120.png      MD787   Mus Musculus    present
MD787-N47-2019.03.28-23.54.40_MD787_2_0140.png      MD787   Mus Musculus    present
MD787-N54-2019.03.29-00.15.46_MD787_1_0160.png      MD787   Mus Musculus    present
MD787-N60-2019.03.29-00.33.42_MD787_3_0180.png      MD787   Mus Musculus    present
MD787-N67-2019.03.29-00.56.05_MD787_2_0200.png      MD787   Mus Musculus    present
MD787-N74-2019.03.29-01.18.34_MD787_1_0220.png      MD787   Mus Musculus    present
MD787-N80-2019.03.29-01.36.50_MD787_3_0240.png      MD787   Mus Musculus    present
MD787-N87-2019.03.29-01.57.37_MD787_2_0260.png      MD787   Mus Musculus    present
MD787-N94-2019.03.29-02.19.41_MD787_1_0280.png      MD787   Mus Musculus    present
MD787-N100-2019.03.29-02.40.34_MD787_3_0300.png     MD787   Mus Musculus    present
MD787-N107-2019.03.29-03.04.17_MD787_2_0320.png     MD787   Mus Musculus    present
MD787-N114-2019.03.29-03.28.07_MD787_1_0340.png     MD787   Mus Musculus    present
MD787-N120-2019.03.29-03.49.00_MD787_3_0360.png     MD787   Mus Musculus    present
```

4.3 Legacy CSV geometry files

In older versions of our pipeline, we stored information in a csv file, with the following 10 fields for each image.

- Filename
- Nx ny nz: number of pixels in the x y and z direction (nz=1 if 2D image)
- Dx dy dz: pixel size in x y and z direction (dz = slice thickness if 2D image)
- X0 y0 z0: coordinate of the first pixel in x y and z direction (z0 = location within dataset if 2D image)

4.4 Cold Spring Harbor legacy geometry files

Cold Spring Harbor is storing geometry data in a plain text file. For example:

```
2021-10-05 14:30:13.177668
Registered : Y
Input Path:/nfs/data/main/M32/RegistrationData/Data_Marmoset/m6344/Transformation_OUTPUT/
↪m6344_img/
Output Path:/nfs/data/main/M32/Cell_Detection/CellDetPass1_reg/m6344/
Number of Files Detected:386
Resolution:0.92
Resolution in Json: 1 micron/pixel
```

4.5 3D imaging data

Our standard is to use simple vtk legacy format for 3D (see https://examples.vtk.org/site/VTKFileFormats/#simple-legacy-formats). Note that this data is always stored in big endian, regardless of machine defaults. These have simple human readable headers that contain the 9 pieces of information above. Our pipeline provides basic support for nifti images using the `nibabel` python package.

4.6 2D microscopy images from Cold Spring Harbor

Acquired microscopy data is stored at Cold Spring Harbor Laboratory in jp2 format at full resolution (generally 0.46 microns per pixel). The filename is generated by the scanner, following a template schema that the Mitra lab uses in a standard manner. An example is:

```
MD787-N3-2019.03.28-21.57.34_MD787_3_0009.jp2
```

Where the meaning of each hyphen separated field is:

```
{sample id}-{N/F/IHC for nissl fluoro or ihc}-{slide number}-{date}-{time}_{sample id}_
↪{what position on slide}_{section number id in anterior to posterior order (generally)}
↪.
```

Note that no geometry information is stored in filenames here, so this should be added as a json companion file.

4.7 2D datasets for registration

Typically data is downsampled by a factor of 32 and saved as a .tif with the same filename.

Registration data can be safely downsampled to approximately the same resolution as atlas images (10-50 micron).

For 2D serial section datasets images should be stored in a single directory using standard imaging formats (i.e. to be read by matplotlib's imread function), downsampled by approximately 32 times (e.g. 14.72 microns for CSH data). While our pipelines do support downsampling to desired resolutions, code will run more efficiently if these sections are already downsampled.

Slice datasets must contain sidecar json files, and data set list tsv files. The script, `histsetup`, generates sidecar files and dataset lists given a subject dataset and voxel spacing (where spacing in the z axis indicates slice thickness plus slice spacing).

4.8 Affine Transformations

Affine transformations are stored as 4x4 matrices written in a text file. Each column is separated by spaces. Each row is separated by a new line. Coordinates are in xyz order. When read into python using our library, they will be converted to zyx order to be consistent with our conventions for indexing image arrays.

4.9 Deformations

Deformations are as 3 component displacement fields (not position fields) in vtk files. In python we work in zyx order, but when writing to vtk fields we switch to xyz order which is the vtk convention.

4.10 Velocity fields

Velocities are nt x 3 component vector fields in vtk files. In python we work in zyx order, but when writing to vtk fields we switch to xyz order which is the vtk convention.

4.11 Annotations

2D annotations are stored as geojson files using the multipolygon data type. Each structure is given a name, and an integer ID. Metadata stores information about which atlas is used, and which 2D image file the annotations correspond to.

These files will also contain atlas coordinate gridlines.

4.12 Point sets

Point sets are stored in vtk polydata format.

4.13 Pixel indexed point sets

Point sets that describe detected cells are stored in geojson format. These point sets have some constraints based on how they will be displayed using open layers or angular on the web.

CHAPTER

FIVE

INPUT SPECIFICATION VIA TRANSFORMATION GRAPH INTERFACE

We support pipelines for registering several datasets to each other, and reconstructing data from one dataset in the space of any other dataset. All of the registrations and reconstructions can be performed by executing a single command from the command line with one input, a json file which contains the following information:

5.1 Names of spaces

Registrations are computed between pairs of spaces. Each space should be given a unique name. (e.g. “atlas”, “CT”, “exvivoMRI”,”invivoMRI”, “Histology”).

5.2 Names of images

Each space may have more than one imaging dataset sampled in it (for example multiple MRI scans with different contrasts). Each image within a space should be given a unique name. (e.g. “exvivoMRI -> T1”, “exvivoMRI -> T2”, “invivoMRI -> T1”, “Histology”)

5.3 Filenames

Each image should have a filename (for 3D data), or a directory (for 2D data) associated with it.

5.4 Registration tuples

To register a complex multimodal dataset, we specify a list of (space/image to map from, space/image to map to) tuples. These correspond to edges in a graph and should span the set of spaces. This set of transformations will be computed using our optimization procedure.

5.5 Registration Configurations

Each registration is computed using unique parameters specified in a registration configuration json file whose path must be listed. These will be loaded into python into a dictionary, which will be passed to functions via keyword arguments. Each registration is run in a multi scale fashion, from low resolution to high resolution, and so each parameter should be specified as a list (one entry for each resolution) or a singleton list (one entry for all resolutions). We have included examples of registration config files in the examples folder.

5.6 Reconstruction tuples

After transformations are computed, we can reconstruct data from one space in any other space. Tuples of the form (space/image to map from, space to map to) are specified. Given the registration tuples, a path of transformations will be computed, which may involve the composition of more than one calculated transform. We can also choose to reconstruct each image in every other space instead of specifying each mapping with a tuple.

5.7 Example

For example we can run registration and reconstruction with the command:

```
python transformation_graph.py --infile INPUT_JSON_FILE
```

Where the input json file contains:

```
{
    "space_image_path": [["MRI", "masked", "/home/brysongray/data/MD816_mini/HR_NIHxCSHL_
↪50um_14T_M1_masked.vtk"],
                        ["CCF", "average_template_50", "/home/brysongray/data/MD816_mini/
↪average_template_50.vtk"],
                        ["MRI", "unmasked", "/home/brysongray/data/MD816_mini/HR_NIHxCSHL_
↪50um_14T_M1.vtk"],
                        ["CT", "masked", "/home/brysongray/data/MD816_mini/ct_mask.vtk"],
                        ["HIST", "nissl", "/home/brysongray/data/MD816_mini/MD816_STIF_
↪mini"]],
    "registrations": [[["MRI", "masked"], ["HIST", "nissl"] ],
                        [ ["CCF", "average_template_50"], ["MRI", "masked"],],
                        [["CT", "masked"], ["MRI", "masked"] ]],
    "configs": ["/home/brysongray/emlddmm/config787small.json",
                "/home/brysongray/emlddmm/configMD816_MR_to_CCF.json",
                "/home/brysongray/emlddmm/configMD816_MR_to_CT.json"],
    "output": "/home/brysongray/emlddmm/transformation_graph_outputs",
    "transforms": [[["CCF", "average_template_50"], ["HIST", "nissl"] ],
                   [["CT", "masked"], ["MRI", "masked"] ]],
    "transform_all": "False"
}
```

This input structure will do the following:

1. It will define 4 spaces, called MRI, CCF, CT and HIST
2. It will define images in these spaces. Paths to images are provided.
   - Two images in the MRI space, called "masked" and "unmasked".

- It will define one image in CCF space called "average_template_50".
- It will define one image in CT space called "masked".
- It will define one image set in HIST space, called "nissl".

3. It will define a set of registrations to calculate. Each registration requires a pair of spaces, and an image name within that space.
    - It will registered the masked MRI to the histology.
    - It will register the CCF atlas to the masked MRI
    - It will register the masked CT to the masked MRI
4. Each registration will be calculated in order, using the config files provided for parameters.
5. Outputs of the registration processes will be saved in the specified output directory.
6. Calculated transforms are applied to map images into new spaces
    - The average template is mapped into the HIST space
    - The masked CT images is mapped into the MRI space.
7. Generally we reconstrct all images in all spaces, in which case transform_all is set to true.

The registration procedure internally sets up the following graph

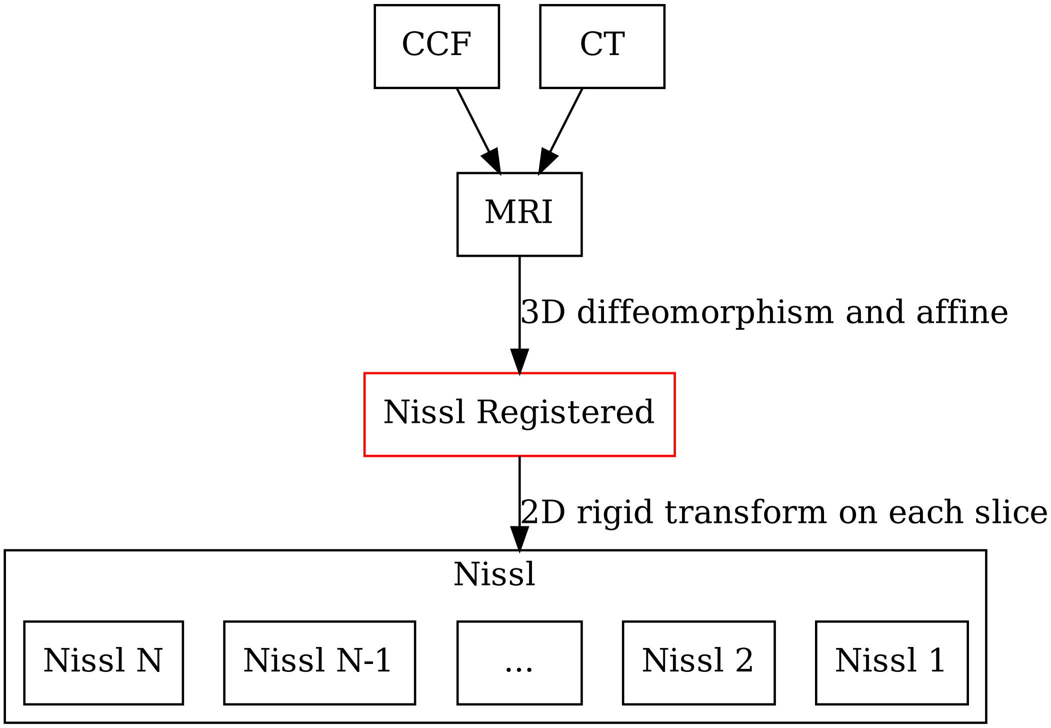

Fig. 1: For any 3D to 2D map, a registered space is automatically created (shown in red). No input data is associated with this space, but images can be reconstructed into this space.

CHAPTER SIX

OUTPUT SPECIFICATION

Our output data structure contains transformations between pairs of named spaces (always), transformed images (suggested but not necessary), and other data types such as points and geojson annotations.

These pairs are organized in a hierarchical tree, where the parent directories contain data in a given space, and the child directories contain data from a given space.

6.1 Example

Example output data structure is shown here. Lists are used to show directory hierarchy: This supports an arbitrary number of folders.:

```
{Space i}
  {Space j}_to_{space i}
    Transforms (always)
      {space i}_to_{space j}_displacement.vtk (3D to 3D, or 3D to registered space, NOT␣
↪3D to input which does not exist as a displacement field)
      {space i}_{image k}_to_{space j}_{image k'}_matrix.txt (2D to 2D only)
      {space i}_{image k}_to_{space j}_displacement.vtk (i 2D to 3D only)
    Images (suggested)
      {space j}_{image k}_to_{space i}.vtk
      {space j}_{image k}_to_{space i}_{image k'}.vtk (for 2D to 2D)
    Points (optional)
      {space j}_{image k}_detects_to_{space i}.vtk
    Json (for atlas only)
      Atlas_to_{space j}_{image k}.geojson
    Meanxyz (for atlas only)
      {space j}_{image k}_detects_to_atlas_meanxyz.txt
  QC (optional)
    Composite_{image slice name}_QC.jpg
```

6.2 Notes

Some important notes are below:

1. Output raster data is stored using simple legacy vtk file format (see *here*).
2. Output point data is stored using simple legacy vtk file format, with polydata.
3. json is shown only for data from atlas to a 2D space.
4. Mean xyz is shown only for a 2D space to the atlas.
5. Transforms are stored as a rigid transformation matrix only for maps from a 2D space to another 2D space.
6. Note the "to" in the naming of transforms is opposite to images. This is intentional.
7. Note that in 2D directories, image names are appended to space names for uniqueness, separated by an underscore.
8. QC figures are not standard, as they will vary by dataset.

CHAPTER SEVEN

EXAMPLES

We include two worked examples in our github repository under the examples folder. In both cases we include an interactive approach that inclues several visualizations, in the form of a jupyter notebook. At the end of each jupyter notebook we show how the same analysis can be run from the command line, using our transformation graph interface, producing our standard output format.

The first describes 3D registration between two human MRI datasets. The second describes 3D to 2D registration between the Allen Nissl atlas, and a sequence of Nissl stained images.

Note that our command line interface has only been validated on Linux systems.

7.1 Human MRI example

In this example we register a pair of 3D human brain MR images.

First we walk through the example in this notebook.

Then we write config files to disk, and run the example from the command line. The command line interface has only been validated on Linux systems.

7.1.1 Import libraries

[1]:

```
# numpy for multidimensional arrays to store images
import numpy as np
# matplotlib for visualization
import matplotlib.pyplot as plt
# the command below will allow interactive figures that update as code runs
%matplotlib notebook

# import tools for working with files
from os import makedirs
from os.path import join

# import the json library for writing out config files
import json

# import the subprocess library for running code from command line
import subprocess

# import the emlddmm image registration library
```

(continues on next page)

(continued from previous page)

```
import sys
sys.path.append('../..')
import emlddmm
```

7.1.2 Outputs

```
[2]: output_directory = 'human_mri_example_notebook_outputs'
```

```
[3]: makedirs(output_directory,exist_ok=True)
```

7.1.3 Load images

```
[4]: target_name = 'TargetMRI.vtk'
atlas_name = 'AtlasMRI.vtk'
label_name = 'AtlasLabels.vtk'
```

```
[5]: # load the atlas with normalization (mean of abs is 1)
xI,I,_,_ = emlddmm.read_data(atlas_name,normalize=True)
# draw a picture
fig,ax = emlddmm.draw(I,xI,cmap='gray')
fig.suptitle('Atlas image')
```

```
<IPython.core.display.Javascript object>
```

```
<IPython.core.display.HTML object>
```

```
[5]: Text(0.5, 0.98, 'Atlas image')
```

```
[6]: # load the atlas segmentation labels, with no normalization (because these are integer␣
↪labels)
xS,S,_,_ = emlddmm.read_data(label_name)
SRGB = emlddmm.labels_to_rgb(S,black_label=256)
# draw a picture, showing labels and MRI
fig,ax = emlddmm.draw(SRGB+I/np.max(I)*2.0,xS)
fig.suptitle('Atlas image')
```

```
<IPython.core.display.Javascript object>
```

```
<IPython.core.display.HTML object>
```

```
[6]: Text(0.5, 0.98, 'Atlas image')
```

```
[7]: # load the target with normalization (mean of abs is 1)
xJ,J,_,_ = emlddmm.read_data(target_name,normalize=True)
# draw a picture
fig,ax = emlddmm.draw(J,xJ,cmap='gray')
fig.suptitle('Target image image')
```

```
<IPython.core.display.Javascript object>
```

```
<IPython.core.display.HTML object>
```

[7]:
```
Text(0.5, 0.98, 'Target image image')
```

[8]:
```
# run registration at 3 different spatial scales
```

[9]:
```
config = {
    'device':'cpu', # cpu or cuda:0
    'downI':[[4,4,4],[2,2,2],[1,1,1]], # downsampling factors for atlas at multiple␣
↪scales
    'downJ':[[4,4,4],[2,2,2],[1,1,1]], # downsampling factors for target at multiple␣
↪scales
    'n_iter':[50,40,30], # how many iterations of gradient descent at each scale
    'v_start':[0], # at what iteration of gradient descent do we start optimizing over␣
↪deformation
    'eA': [1e1], # gradient descent stepsize for 3D affine transform
    'ev':[5e-1], # gradient descent stepsize for the deformation
    'a':2.0, # spatial scale of deformation
    'dv':2.0, # sampling interval for deformation
    'sigmaR':5e0, # regularizatoin for deformation (bigger means less regularization)
    'local_contrast':[[32,32,32]] # divide the images into small blocks to estimate␣
↪contrast differences
}
```

[10]:
```
out = emlddmm.emlddmm_multiscale(xI=[xI],I=I,xJ=[xJ],J=J,**config)
```

```
Found 3 scales
```

```
<IPython.core.display.Javascript object>
```

```
<IPython.core.display.HTML object>
```

```
<IPython.core.display.Javascript object>
```

```
<IPython.core.display.HTML object>
```

```
<IPython.core.display.Javascript object>
```

```
<IPython.core.display.HTML object>
```

```
<IPython.core.display.Javascript object>
```

```
<IPython.core.display.HTML object>
```

```
<IPython.core.display.Javascript object>
```

```
<IPython.core.display.HTML object>
```

```
<IPython.core.display.Javascript object>
```

```
<IPython.core.display.HTML object>
```

```
<IPython.core.display.Javascript object>
```

```
<IPython.core.display.HTML object>
```

```
<IPython.core.display.Javascript object>
```

```
<IPython.core.display.HTML object>
```

```
../../emlddmm.py:175: RuntimeWarning: invalid value encountered in true_divide
  J /= (vmax[:,None,None,None] - vmin[:,None,None,None])
```

```
Iteration 30, linear oscilating, reducing eA to 9.0
Iteration 40, translation oscilating, reducing eA to 8.1
```

```
../../emlddmm.py:1334: UserWarning: To copy construct from a tensor, it is recommended␣
↪to use sourceTensor.clone().detach() or sourceTensor.clone().detach().requires_grad_
↪(True), rather than torch.tensor(sourceTensor).
  v = torch.tensor(v.detach().clone(),device=device,dtype=dtype)
../../emlddmm.py:1378: UserWarning: To copy construct from a tensor, it is recommended␣
↪to use sourceTensor.clone().detach() or sourceTensor.clone().detach().requires_grad_
↪(True), rather than torch.tensor(sourceTensor).
  A = torch.tensor(A.detach().clone(),device=device,dtype=dtype)
```

```
<IPython.core.display.Javascript object>
```

```
<IPython.core.display.HTML object>
```

```
<IPython.core.display.Javascript object>
```

```
<IPython.core.display.HTML object>
```

```
<IPython.core.display.Javascript object>
```

```
<IPython.core.display.HTML object>
```

```
<IPython.core.display.Javascript object>
```

```
<IPython.core.display.HTML object>
```

```
<IPython.core.display.Javascript object>
```

```
<IPython.core.display.HTML object>
```

```
<IPython.core.display.Javascript object>
```

```
<IPython.core.display.HTML object>
```

```
<IPython.core.display.Javascript object>
```

```
<IPython.core.display.HTML object>
```

```
<IPython.core.display.Javascript object>
```

```
<IPython.core.display.HTML object>
```

```
<IPython.core.display.Javascript object>
```

```
<IPython.core.display.HTML object>
```

```
../../emlddmm.py:1445: RuntimeWarning: More than 20 figures have been opened. Figures␣
↪created through the pyplot interface (`matplotlib.pyplot.figure`) are retained until␣
↪explicitly closed and may consume too much memory. (To control this warning, see the␣
↪rcParam `figure.max_open_warning`).
  figA,axA = plt.subplots(2,2)
```

```
<IPython.core.display.Javascript object>
```

```
<IPython.core.display.HTML object>
```

```
<IPython.core.display.Javascript object>
```

```
<IPython.core.display.HTML object>
```

```
<IPython.core.display.Javascript object>
```

```
<IPython.core.display.HTML object>
```

```
<IPython.core.display.Javascript object>
```

```
<IPython.core.display.HTML object>
```

```
<IPython.core.display.Javascript object>
```

```
<IPython.core.display.HTML object>
```

```
<IPython.core.display.Javascript object>
```

```
<IPython.core.display.HTML object>
```

```
<IPython.core.display.Javascript object>
```

```
<IPython.core.display.HTML object>
```

7.1.4 apply the transform to the atlas image

We use the backward (inverse) transformations.

[11]:
```
tform = emlddmm.compose_sequence(
    [
        emlddmm.Transform(out[-1]['A'],direction='b'),
        emlddmm.Transform(out[-1]['v'],domain=out[-1]['xv'],direction='b')
    ],
    xJ
)
AphiI = emlddmm.apply_transform_float(xI,I,tform)
AphiS = emlddmm.apply_transform_int(xS,S,tform)
```

[12]:
```
# draw the labels over the target image
AphiSRGB = emlddmm.labels_to_rgb(AphiS,black_label=256)
# draw a picture, showing labels and MRI
fig,ax = emlddmm.draw(AphiSRGB+J/np.max(J)*2.0,xJ)
fig.suptitle('Target image with atlas labels')
```

```
<IPython.core.display.Javascript object>
```

```
<IPython.core.display.HTML object>
```

[12]:
```
Text(0.5, 0.98, 'Target image with atlas labels')
```

[13]:
```
# save the transformed images
emlddmm.write_data(join(output_directory,'atlas_labels_to_target.vtk'),xJ,AphiS,title=
↪'atlas_labels_to_target')
emlddmm.write_data(join(output_directory,'atlas_image_to_target.vtk'),xJ,AphiI,title=
↪'atlas_image_to_target')
```

7.1.5 apply the transform to the target image

We use the forward transformation

[14]:
```
# transform target back to template
tform = emlddmm.compose_sequence(
    [
        emlddmm.Transform(out[-1]['v'],domain=out[-1]['xv'],direction='f'),
        emlddmm.Transform(out[-1]['A'],direction='f'),
    ],
    xI
)
phiiAiJ = emlddmm.apply_transform_float(xJ,J,tform)

# resample target on template voxels
tform = emlddmm.compose_sequence([emlddmm.Transform(np.eye(4))],xI)
Jresampled = emlddmm.apply_transform_float(xJ,J,tform)
```

[15]:
```
fig,ax = emlddmm.draw(np.concatenate((I,Jresampled)),xI)
fig.suptitle('Atlas in magenta, UNtransformed target in green')

fig,ax = emlddmm.draw(np.concatenate((I,phiiAiJ)),xI)
fig.suptitle('Atlas in magenta, transformed target in green')
```

```
<IPython.core.display.Javascript object>
```

```
<IPython.core.display.HTML object>
```

```
<IPython.core.display.Javascript object>
```

```
<IPython.core.display.HTML object>
```

[15]:
```
Text(0.5, 0.98, 'Atlas in magenta, transformed target in green')
```

[16]:
```
# save the transformed image
emlddmm.write_data(join(output_directory,'target_image_to_atlas.vtk'),xJ,AphiS,title=
↪'target_image_to_atlas')
```

7.1.6 Run the same analysis using the command line interface

Next we will show how to produce necessary config files, and run the same example using our transformation graph command line interface.

7.1.7 write out a config file for registration

[17]:
```
config_file = 'atlas_to_target_config.json'
with open(config_file,'wt') as f:
    json.dump(config,f)
```

7.1.8 write out a config file for the transformation graph

[18]:
```
transformation_config = {
  "output": "output",
  "space_image_path": [
    [
      "Atlas",
      "image",
      atlas_name
    ],
    [
      "Atlas",
      "labels",
      label_name
    ],
    [
      "Target",
      "image",
      target_name
    ],
  ],
  "registrations": [
    [
      [
        "Atlas",
        "image"
      ],
      [
        "Target",
        "image"
      ]
    ],
  ],
  "configs": [
    config_file,

  ],
  "transform_all":True,
}
transformation_config_file = 'transformation_graph_config.json'
with open(transformation_config_file,'wt') as f:
    json.dump(transformation_config,f)
```

7.1.9 Run this example from the command line

We will write out the parameters above to a config file, and run our command line interface. This will produce all our standard outputs.

```
[19]: command = f'python -u ../../transformation_graph_v01.py --infile {transformation_config_
      ↪file} > outputs.txt 2>&1'
      print('about to run command:')
      print(command)
```

```
about to run command:
python -u ../../transformation_graph_v01.py --infile transformation_graph_config.json >␣
↪outputs.txt 2>&1
```

```
[20]: subprocess.call(command,shell=True)
```

```
[20]: 0
```

7.1.10 View all the outputs

All the output directories are printed below. Note that this includes a python file graph.p, which contains information about the transformation graph. This will allow us to apply the transforms we have already calculated to new datasets later.

```
[27]: from os import walk
      for dirpath,dirnames,filenames in walk('output',):
          for f in filenames:
              print(join(dirpath,f))
```

```
output/infile.json
output/graph.p
output/Atlas/Target_to_Atlas/transforms/velocity.vtk
output/Atlas/Target_to_Atlas/transforms/A.txt
output/Atlas/Target_to_Atlas/transforms/Target_to_Atlas_displacement.vtk
output/Atlas/Target_to_Atlas/transforms/Target_image_to_Atlas_detjac.vtk
output/Atlas/Target_to_Atlas/qc/Target_image_to_Atlas.jpg
output/Atlas/Target_to_Atlas/qc/Atlas_image.jpg
output/Atlas/Target_to_Atlas/images/Target_image_to_Atlas.vtk
output/Target/Atlas_to_Target/qc/Atlas_image_to_Target.jpg
output/Target/Atlas_to_Target/qc/Target_image.jpg
output/Target/Atlas_to_Target/images/Atlas_image_to_Target.vtk
output/Target/Atlas_to_Target/images/Atlas_labels_to_Target.vtk
output/Target/Atlas_to_Target/transforms/Atlas_to_Target_displacement.vtk
output/Target/Atlas_to_Target/transforms/Atlas_image_to_Target_detjac.vtk
output/Target/Atlas_to_Target/transforms/Atlas_labels_to_Target_detjac.vtk
```

7.2 Mouse Serial Section Example

In this example we will register the Allen CCF atlas to a mouse Nissl dataset.

First we will walk through an exapmle in this notebook.

Then we write config files to disk, and run the example from the command line. The command line interface has only been validated on Linux systems.

7.2.1 Import libraries

[1]:
```
# numpy for multidimensional arrays to store images
import numpy as np
# matplotlib for visualization
import matplotlib.pyplot as plt
# the command below will allow interactive figures that update as code runs
%matplotlib notebook

# import tools for working with files
from os import makedirs
from os.path import join

# import the json library for writing out config files
import json

# import the subprocess library for running code from command line
import subprocess

# import the emlddmm image registration library
import sys
sys.path.append('../..')
import emlddmm
```

7.2.2 Load images

[2]:
```
target_name = '/home/dtward/data/csh_data/emlddmm/mouse_example/NisslDown/'
atlas_name = '/home/dtward/data/AllenInstitute/allen_vtk/ara_nissl_50.vtk'
label_name = '/home/dtward/data/AllenInstitute/allen_vtk/annotation_50.vtk'
```

[3]:
```
# load the atlas with normalization (mean of abs is 1)
xI,I,_,_ = emlddmm.read_data(atlas_name,normalize=True)
# draw a picture
fig,ax = emlddmm.draw(I,xI,cmap='gray')
fig.suptitle('Atlas image')
```

```
<IPython.core.display.Javascript object>
```

```
<IPython.core.display.HTML object>
```

[3]:
```
Text(0.5, 0.98, 'Atlas image')
```

[4]:
```
# load the atlas segmentation labels, with no normalization (because these are integer␣
↪labels)
xS,S,_,_ = emlddmm.read_data(label_name)
SRGB = emlddmm.labels_to_rgb(S)
# draw a picture, showing labels and MRI
fig,ax = emlddmm.draw(SRGB+I/np.max(I)*2.0,xS)
fig.suptitle('Atlas image')
```

```
<IPython.core.display.Javascript object>
```

```
<IPython.core.display.HTML object>
```

[4]:
```
Text(0.5, 0.98, 'Atlas image')
```

[5]:
```
# load the target
xJ,J,_,_ = emlddmm.read_data(target_name)
# "weights" for missing data are stored in the last channel
W = J[-1]
J = J[:-1]
# draw a picture
fig,ax = emlddmm.draw(J,xJ,cmap='gray')
fig.suptitle('Target image image')
```

```
<IPython.core.display.Javascript object>
```

```
<IPython.core.display.HTML object>
```

```
<IPython.core.display.Javascript object>
```

```
<IPython.core.display.HTML object>
```

[5]:
```
Text(0.5, 0.98, 'Target image image')
```

7.2.3 Perform an initial "slice to neighbor" alignment

[6]:
```
# downsample, to speed up calculations
xJd,Jd,Wd = emlddmm.downsample_image_domain(xJ,J,[1,2,2],W=W)
emlddmm.draw(Jd,xJd,vmin=0,vmax=1,interpolation='none')
```

```
<IPython.core.display.Javascript object>
```

```
<IPython.core.display.HTML object>
```

[6]:
```
(<Figure size 640x480 with 15 Axes>,
 array([[<AxesSubplot:>, <AxesSubplot:>, <AxesSubplot:>, <AxesSubplot:>,
         <AxesSubplot:>],
        [<AxesSubplot:>, <AxesSubplot:>, <AxesSubplot:>, <AxesSubplot:>,
         <AxesSubplot:>],
        [<AxesSubplot:>, <AxesSubplot:>, <AxesSubplot:>, <AxesSubplot:>,
         <AxesSubplot:>]], dtype=object))
```

[7]:
```
# run slice to neighbor alignment
# it gave terrible results!
# everything seemed to shift out of frame
import importlib
importlib.reload(emlddmm)
```

(continues on next page)

(continued from previous page)

```
out0 = emlddmm.atlas_free_reconstruction(xJ=xJd,J=Jd,W=(Wd==1),draw=True,n_steps=10,
↪eA2d=2e4)
```

```
../../emlddmm.py:4984: RuntimeWarning: divide by zero encountered in true_divide
  op = 1.0 / (xJ[0] - xJ[0][0])
../../emlddmm.py:4986: RuntimeWarning: divide by zero encountered in true_divide
  op = 1.0 / (xJ[0] - xJ[0][0])**2
../../emlddmm.py:4987: RuntimeWarning: divide by zero encountered in true_divide
  op = 1.0 / np.abs((xJ[0] - xJ[0][0]))**1.5/np.sign((xJ[0] - xJ[0][0]))
```

```
<IPython.core.display.Javascript object>
```

```
<IPython.core.display.HTML object>
```

```
<IPython.core.display.Javascript object>
```

```
<IPython.core.display.HTML object>
```

```
<IPython.core.display.Javascript object>
```

```
<IPython.core.display.HTML object>
```

```
<IPython.core.display.Javascript object>
```

```
<IPython.core.display.HTML object>
```

```
starting it 0
```

```
/home/dtward/.local/intelpython3/lib/python3.7/site-packages/torch/autograd/__init__.py:
↪199: UserWarning: grad and param do not obey the gradient layout contract. This is not␣
↪an error, but may impair performance.
grad.sizes() = [668, 3, 3], strides() = [9, 3, 1]
param.sizes() = [668, 3, 3], strides() = [9, 1, 3] (Triggered internally at ../torch/
↪csrc/autograd/functions/accumulate_grad.h:202.)
  allow_unreachable=True, accumulate_grad=True)  # Calls into the C++ engine to run the␣
↪backward pass
```

```
starting it 1
```

```
../../emlddmm.py:1404: UserWarning: To copy construct from a tensor, it is recommended␣
↪to use sourceTensor.clone().detach() or sourceTensor.clone().detach().requires_grad_
↪(True), rather than torch.tensor(sourceTensor).
  A2d = torch.tensor(A2d.detach().clone(),device=device, dtype=dtype)
```

```
starting it 2
starting it 3
starting it 4
starting it 5
starting it 6
starting it 7
starting it 8
starting it 9
```

7.2.4 Run atlas to slice alignment

[8]:
```
config = {
    'device':'cpu', # cpu or cuda:0
    'downI':[[4,4,4],[2,2,2],[1,1,1]], # downsampling factors for the atlas for multi␣
↪scale
    'downJ':[[1,4,4],[1,2,2],[1,1,1]], # downsampling factors for the target for multi␣
↪scale. don't downsample in the slice direction (first number)
    'n_iter':[100,50,25], # number of iterations of gradient descent
    'a':[500.0], # spatial scale of the deformation
    'dv':[1000.0], # voxel size to sample the deformation on
    'muB':[[1.0,1.0,1.0]], # estimate of the intensity of the background (white)
    'muA':[[0.0,0.0,0.0]], # estimate of the intensitiy of artifacts (black)
    'slice_matching':[True], # enable rigid motions over slices
    'v_start':[0], # at which iteration do we start optimizing over the deformation
    'eA':[1e7], # gradient descent stepsize for 3D affine
    'eA2d':[1e5], # gradient descent stepsize for 2D rigid
    'ev':[1e-2], # gradient descent stepsize for deformation
    'local_contrast':[[1,16,16]], # divide the images into small blocks to estimate␣
↪contrast differences
    'up_vector':[[0.0,0.0,-1.0]], # what vector in the atlas should correspond to "up"␣
↪in a 2D image
    'sigmaR':[1e4] # regularization for deformation (bigger = less regularization)
}
# intial 2D alignment
config['A2d'] = out0['A2d']
# initial 3D affine
# look at the above figures and assin a letter to each row in order
# A means this row moves from posterior to anterior
# P means this row moves from anterior to posterior
# S means this row moves from inferior to superior
# I means this row moves from superior to inferior
# R means this row moves from left to right
# L means this row moves from right to left
# note that often it is difficult to tell left from right, so we always assume a right␣
↪handed coordinates
A = np.eye(4)
A[:3,:3] = emlddmm.orientation_to_orientation('ARI','PSL')
config['A'] = A
```

[9]:
```
import time
start = time.time()
import importlib
importlib.reload(emlddmm)
out = emlddmm.emlddmm_multiscale(xI=[xI],I=I,xJ=[xJ],J=J,W0=W,**config)
end = time.time()
print(end-start) # print out the elapsed time, which was reported in the manuscript
```

```
Found 3 scales
```

```
<IPython.core.display.Javascript object>
```

```
<IPython.core.display.HTML object>
```

```
<IPython.core.display.Javascript object>
```

```
<IPython.core.display.HTML object>
```

```
<IPython.core.display.Javascript object>
```

```
<IPython.core.display.HTML object>
```

```
<IPython.core.display.Javascript object>
```

```
<IPython.core.display.HTML object>
```

```
<IPython.core.display.Javascript object>
```

```
<IPython.core.display.HTML object>
```

```
<IPython.core.display.Javascript object>
```

```
<IPython.core.display.HTML object>
```

```
<IPython.core.display.Javascript object>
```

```
<IPython.core.display.HTML object>
```

```
<IPython.core.display.Javascript object>
```

```
<IPython.core.display.HTML object>
```

```
<IPython.core.display.Javascript object>
```

```
<IPython.core.display.HTML object>
```

```
../../emlddmm.py:176: RuntimeWarning: invalid value encountered in true_divide
  J /= (vmax[:,None,None,None] - vmin[:,None,None,None])
```

```
Iteration 70, linear oscilating, reducing eA to 9000000.0
Iteration 80, linear oscilating, reducing eA to 8100000.0
```

```
../../emlddmm.py:1335: UserWarning: To copy construct from a tensor, it is recommended␣
↪to use sourceTensor.clone().detach() or sourceTensor.clone().detach().requires_grad_
↪(True), rather than torch.tensor(sourceTensor).
  v = torch.tensor(v.detach().clone(),device=device,dtype=dtype)
../../emlddmm.py:1379: UserWarning: To copy construct from a tensor, it is recommended␣
↪to use sourceTensor.clone().detach() or sourceTensor.clone().detach().requires_grad_
↪(True), rather than torch.tensor(sourceTensor).
  A = torch.tensor(A.detach().clone(),device=device,dtype=dtype)
```

```
<IPython.core.display.Javascript object>
```

```
<IPython.core.display.HTML object>
```

```
<IPython.core.display.Javascript object>
```

```
<IPython.core.display.HTML object>
```

```
../../emlddmm.py:1449: RuntimeWarning: More than 20 figures have been opened. Figures␣
↪created through the pyplot interface (`matplotlib.pyplot.figure`) are retained until␣
↪explicitly closed and may consume too much memory. (To control this warning, see the␣
↪rcParam `figure.max_open_warning`).
  figA2d,axA2d = plt.subplots(2,2)
```

```
<IPython.core.display.Javascript object>
```

```
<IPython.core.display.HTML object>
```

```
<IPython.core.display.Javascript object>
```

```
<IPython.core.display.HTML object>
```

```
<IPython.core.display.Javascript object>
```

```
<IPython.core.display.HTML object>
```

```
<IPython.core.display.Javascript object>
```

```
<IPython.core.display.HTML object>
```

```
<IPython.core.display.Javascript object>
```

```
<IPython.core.display.HTML object>
```

```
<IPython.core.display.Javascript object>
```

```
<IPython.core.display.HTML object>
```

```
<IPython.core.display.Javascript object>
```

```
<IPython.core.display.HTML object>
```

```
Iteration 20, linear oscilating, reducing eA to 9000000.0
Iteration 40, linear oscilating, reducing eA to 8100000.0
```

```
<IPython.core.display.Javascript object>
```

```
<IPython.core.display.HTML object>
```

```
<IPython.core.display.Javascript object>
```

```
<IPython.core.display.HTML object>
```

```
<IPython.core.display.Javascript object>
```

```
<IPython.core.display.HTML object>
```

```
<IPython.core.display.Javascript object>
```

```
<IPython.core.display.HTML object>
```

```
<IPython.core.display.Javascript object>
```

```
<IPython.core.display.HTML object>
```

```
<IPython.core.display.Javascript object>
```

```
<IPython.core.display.HTML object>
```

```
<IPython.core.display.Javascript object>
```

```
<IPython.core.display.HTML object>
```

```
<IPython.core.display.Javascript object>
```

```
<IPython.core.display.HTML object>
```

```
<IPython.core.display.Javascript object>
```

```
<IPython.core.display.HTML object>
```

```
715.610100030899
```

7.2.5 Apply the transform from atlas to registered space

We use the inverse transform.

[10]:
```
tform = emlddmm.compose_sequence(
    [
        emlddmm.Transform(out[-1]['A'],direction='b'),
        emlddmm.Transform(out[-1]['v'],domain=out[-1]['xv'],direction='b'),
    ],
    xJ
)
AphiI = emlddmm.apply_transform_float(xI,I,tform)
AphiS = emlddmm.apply_transform_int(xS,S,tform)
```

7.2.6 Apply the transform from target to registered space

We use the forward transform

[11]:
```
tform = emlddmm.compose_sequence(
    [
        emlddmm.Transform(out[-1]['A2d'],direction='f'),
    ],
    xJ
)
RiJ = emlddmm.apply_transform_float(xJ,J,tform)
```

[12]:
```
# draw the labels over the target image
AphiSRGB = emlddmm.labels_to_rgb(AphiS,white_label=0)
# draw a picture, showing labels and MRI
fig,ax = emlddmm.draw(AphiSRGB*0.125+RiJ.numpy()/RiJ.max().numpy()*0.875,xJ)
fig.suptitle('Target image with atlas labels')
```

```
<IPython.core.display.Javascript object>
```

```
<IPython.core.display.HTML object>
```

[12]:
```
Text(0.5, 0.98, 'Target image with atlas labels')
```

7.2.7 Apply the transform from registered target to atlas space

Interpolation between slices is only meaningful after 2D alignment. Here we apply a transformation to our aligned Nissl slices.

[24]:
```
tform = emlddmm.compose_sequence(
    [
        emlddmm.Transform(out[-1]['v'],domain=out[-1]['xv'],direction='f'),
        emlddmm.Transform(out[-1]['A'],direction='f'),
    ],
    xI
)
phiiAiRiJ = emlddmm.apply_transform_float(xJ,RiJ,tform)
```

[27]:
```
fig,ax = emlddmm.draw(phiiAiRiJ)
```

```
<IPython.core.display.Javascript object>
```

```
<IPython.core.display.HTML object>
```

7.2.8 Run this example from the command line

We will write out the parameters above to a config file, and run our command line interface. This will produce all our standard outputs.

7.2.9 Write out the registration config file

We'll make sure some of the config inputs are formatted properly as plain text for our config file, and write it out..

[13]:
```
config_ = dict(config)
config_['A2d'] = [config_['A2d'].tolist()]
config_['A'] = [config_['A'].tolist()]
```

[14]:
```
config_file = 'atlas_to_target_config.json'
with open(config_file,'wt') as f:
    json.dump(config_,f)
```

7.2.10 Write out the transformation graph config file

[15]:
```
transformation_config = {
  "output": "output",
  "space_image_path": [
    [
      "Atlas",
      "image",
      atlas_name
    ],
    [
      "Atlas",
      "labels",
      label_name
    ],
    [
      "Target",
      "image",
      target_name
    ],
  ],
  "registrations": [
    [
      [
        "Atlas",
        "image"
      ],
```

(continues on next page)

(continued from previous page)

```
      [
        "Target",
        "image"
      ]
    ],
  ],
  "configs": [
    config_file,

  ],
  "transform_all":True,
}
transformation_config_file = 'transformation_graph_config.json'
with open(transformation_config_file,'wt') as f:
    json.dump(transformation_config,f)
```

7.2.11 Run the command

[16]:
```
command = f'python -u ../../transformation_graph_v01.py --infile {transformation_config_
↪file} > outputs.txt 2>&1'
print('about to run command:')
print(command)
```

```
about to run command:
python -u ../../transformation_graph_v01.py --infile transformation_graph_config.json >␣
↪outputs.txt 2>&1
```

[17]:
```
subprocess.call(command,shell=True)
```

[17]:
```
0
```

7.2.12 View all the outputs

All the output directories are printed below. Note that this includes a python file graph.p, which contains information about the transformation graph. This will allow us to apply the transforms we have already calculated to new datasets later.

[20]:
```
from os import walk
for dirpath,dirnames,filenames in walk('output',):
    for i,f in enumerate(filenames):
        if i >= 3:
            print('...more files for each slice...')
            break
        print(join(dirpath,f))
```

```
output/infile.json
output/graph.p
output/Target_registered/Target_to_Target_registered/transforms/Target_registered_PTM902-
↪N1-2021.05.27-15.39.29_PTM902_3_0001_to_Target_PTM902-N1-2021.05.27-15.39.29_PTM902_3_
↪0001_matrix.txt
```

(continues on next page)

(continued from previous page)

```
output/Target_registered/Target_to_Target_registered/transforms/Target_registered_PTM902-
↪N1-2021.05.27-15.39.29_PTM902_2_0002_to_Target_PTM902-N1-2021.05.27-15.39.29_PTM902_2_
↪0002_matrix.txt
output/Target_registered/Target_to_Target_registered/transforms/Target_registered_PTM902-
↪N1-2021.05.27-15.39.29_PTM902_1_0003_to_Target_PTM902-N1-2021.05.27-15.39.29_PTM902_1_
↪0003_matrix.txt
...more files for each slice...
output/Target_registered/Target_to_Target_registered/images/Target_image_PTM902-N1-2021.
↪05.27-15.39.29_PTM902_3_0001_to_Target_registered_PTM902-N1-2021.05.27-15.39.29_PTM902_
↪3_0001.vtk
output/Target_registered/Target_to_Target_registered/images/Target_image_PTM902-N1-2021.
↪05.27-15.39.29_PTM902_2_0002_to_Target_registered_PTM902-N1-2021.05.27-15.39.29_PTM902_
↪2_0002.vtk
output/Target_registered/Target_to_Target_registered/images/Target_image_PTM902-N1-2021.
↪05.27-15.39.29_PTM902_1_0003_to_Target_registered_PTM902-N1-2021.05.27-15.39.29_PTM902_
↪1_0003.vtk
...more files for each slice...
output/Target_registered/Atlas_to_Target_registered/qc/Target_image_registered.jpg
output/Target_registered/Atlas_to_Target_registered/qc/Atlas_image_to_Target_registered.
↪jpg
output/Target_registered/Atlas_to_Target_registered/images/Atlas_image_to_Target_
↪registered_PTM902-N1-2021.05.27-15.39.29_PTM902_3_0001.vtk
output/Target_registered/Atlas_to_Target_registered/images/Atlas_image_to_Target_
↪registered_PTM902-N1-2021.05.27-15.39.29_PTM902_2_0002.vtk
output/Target_registered/Atlas_to_Target_registered/images/Atlas_image_to_Target_
↪registered_PTM902-N1-2021.05.27-15.39.29_PTM902_1_0003.vtk
...more files for each slice...
output/Target_registered/Atlas_to_Target_registered/transforms/Target_registered_PTM902-
↪N1-2021.05.27-15.39.29_PTM902_3_0001_to_Atlas_displacement.vtk
output/Target_registered/Atlas_to_Target_registered/transforms/Target_registered_PTM902-
↪N1-2021.05.27-15.39.29_PTM902_2_0002_to_Atlas_displacement.vtk
output/Target_registered/Atlas_to_Target_registered/transforms/Target_registered_PTM902-
↪N1-2021.05.27-15.39.29_PTM902_1_0003_to_Atlas_displacement.vtk
...more files for each slice...
output/Atlas/Target_registered_to_Atlas/transforms/velocity.vtk
output/Atlas/Target_registered_to_Atlas/transforms/A.txt
output/Atlas/Target_registered_to_Atlas/transforms/Atlas_to_Target_registered_
↪displacement.vtk
output/Atlas/Target_registered_to_Atlas/qc/Target_image_to_Atlas.jpg
output/Atlas/Target_registered_to_Atlas/qc/Atlas_image.jpg
output/Atlas/Target_to_Atlas/images/Target_image_to_Atlas.vtk
output/Atlas/Target_to_Atlas/transforms/Atlas_to_Target_displacement.vtk
output/Target/Atlas_to_Target/qc/Atlas_image_to_Target.jpg
output/Target/Atlas_to_Target/qc/Target_image.jpg
output/Target/Atlas_to_Target/images/Atlas_image_to_Target_PTM902-N1-2021.05.27-15.39.29_
↪PTM902_3_0001.vtk
output/Target/Atlas_to_Target/images/Atlas_image_to_Target_PTM902-N1-2021.05.27-15.39.29_
↪PTM902_2_0002.vtk
output/Target/Atlas_to_Target/images/Atlas_image_to_Target_PTM902-N1-2021.05.27-15.39.29_
↪PTM902_1_0003.vtk
...more files for each slice...
output/Target/Atlas_to_Target/transforms/Target_PTM902-N1-2021.05.27-15.39.29_PTM902_3_
```

(continues on next page)

(continued from previous page)

```
↪0001_to_Atlas_displacement.vtk
output/Target/Atlas_to_Target/transforms/Target_PTM902-N1-2021.05.27-15.39.29_PTM902_2_
↪0002_to_Atlas_displacement.vtk
output/Target/Atlas_to_Target/transforms/Target_PTM902-N1-2021.05.27-15.39.29_PTM902_1_
↪0003_to_Atlas_displacement.vtk
...more files for each slice...
output/Target/Target_registered_to_Target/transforms/Target_PTM902-N1-2021.05.27-15.39.
↪29_PTM902_3_0001_to_Target_registered_PTM902-N1-2021.05.27-15.39.29_PTM902_3_0001_
↪matrix.txt
output/Target/Target_registered_to_Target/transforms/Target_PTM902-N1-2021.05.27-15.39.
↪29_PTM902_2_0002_to_Target_registered_PTM902-N1-2021.05.27-15.39.29_PTM902_2_0002_
↪matrix.txt
output/Target/Target_registered_to_Target/transforms/Target_PTM902-N1-2021.05.27-15.39.
↪29_PTM902_1_0003_to_Target_registered_PTM902-N1-2021.05.27-15.39.29_PTM902_1_0003_
↪matrix.txt
...more files for each slice...
```

CHAPTER

EIGHT

FUNCTION REFERENCE

CHAPTER

NINE

WORK IN PROGRESS

This section contains a description of works in progress.

9.1 Blockface imaging

Our pipeline supports a sequence of blockface photographs for 3D reconstruction rather than a tranditional 3D volume. We will prepare examples related to this application.

CHAPTER

TEN

INSTALLING

- Make sure you have python 3 installed with pip
- Use pip to install the packages in the requirements.txt file (pip in stall -r requrements.txt)
- Clone the repository on github (git clone https://github.com/twardlab/emlddmm)
- When running interactively in python, make sure you add the path (import sys; sys.path.append('/LOCATION/OF/REPOSITORY'))

CHAPTER

ELEVEN

EXAMPLES

We include data and code for two examples, in the "examples" folder. Both examples show code run interactively in a jupyter notebook, and show how the command line interface is used.

- 3D Human MRI example
- Mouse Nissl serial section alignment example.

CHAPTER

TWELVE

IMPORTANT FUNCTIONS

12.1 Python functions to be run interactively

- `emlddmm.emlddmm()`: Run the emlddmm algorithm.
- `emlddmm.emlddmm_multiscale()`: Run the emlddmm algorithm iteratively at different scales.

12.2 Command line functions

- `transformation_graph_v01`: Run registration between two or more datasets using the transformation graph command line interface.

CHAPTER

THIRTEEN

MODULE AND FUNCTION DOCUMENTATION

All functions are automatically documented with sphinx and napoleon. See the Function Reference section.

CHAPTER FOURTEEN

WEB INTERFACE

To improve accessibility, we provide a web interface at https://twardlab.com/reg which can be used for small jobs. Please email the Daniel Tward (dtward@mednet.ucla.edu) to request an account. We provide a guest account for review purposes. Username: guest, password: 84983c60. No identifying information is recorded in the guest account. We request email addresses when creating your own account.
