# Supplementary material for: Solving the where problem and quantifying geometric variation in neuroanatomy using generative diffeomorphic mapping
Source: Nat Commun. 2025 Nov 24;16:10398. doi: 10.1038/s41467-025-65317-7 (PMC12645051; doi:10.1038/s41467-025-65317-7)
Supplement: Supplementary file 2 — Description of Additional Supplementary Files [file 41467_2025_65317_MOESM2_ESM.pdf]

Description of Additional Supplementary Files

Title: Supplementary Data 1.
Description: Figshare https://doi.org/10.6084/m9.figshare.25106168 , PVdensities.csv. This file contains mean and standard deviation of our estimates of cell density for PV Cells in 794 annotated atlas regions.

Title: Supplementary Data 2.
Description: Figshare https://doi.org/10.6084/m9.figshare.25106168 , SOMdensities.csv. This file contains mean and standard deviation of our estimates of cell density for SOM Cells in 794 annotated atlas regions.

Title: Supplementary Data 3.
Description: Figshare https://doi.org/10.6084/m9.figshare.25106168 , VIPdensities.csv. This file contains mean and standard deviation of our estimates of cell density for VIP Cells in 794 annotated atlas regions.

Title: Supplementary Data 4.
Description: Figshare https://doi.org/10.6084/m9.figshare.25106168 , density_volumes. This folder contains 27 density images (i.e. a density value at each voxel) in vtk format. The specific cell label, age, and sex of the corresponding mouse are indicated in each filename.
